# Liver regeneration after injury: Mechanisms, cellular interactions and therapeutic innovations

**DOI:** 10.1002/ctm2.1812

**Published:** 2024-08-16

**Authors:** Qi Liu, Senyan Wang, Jing Fu, Yao Chen, Jing Xu, Wenjuan Wei, Hao Song, Xiaofang Zhao, Hongyang Wang

**Affiliations:** ^1^ Translational Medicine Centre The First Affiliated Hospital of Zhengzhou University Zhengzhou Henan Province China; ^2^ Department of Hepatobiliary and Pancreatic Surgery The First Affiliated Hospital of Zhengzhou University Zhengzhou Henan Province China; ^3^ International Cooperation Laboratory on Signal Transduction National Center for Liver Cancer Ministry of Education Key Laboratory on Signaling Regulation and Targeting Therapy of Liver Cancer Shanghai Key Laboratory of Hepato‐biliary Tumor Biology Eastern Hepatobiliary Surgery Hospital, Second Military Medical University/NAVAL Medical University Shanghai China

**Keywords:** interventions, liver injury model, liver regeneration, signalling pathway

## Abstract

The liver possesses a distinctive capacity for regeneration within the human body. Under normal circumstances, liver cells replicate themselves to maintain liver function. Compensatory replication of healthy hepatocytes is sufficient for the regeneration after acute liver injuries. In the late stage of chronic liver damage, a large number of hepatocytes die and hepatocyte replication is blocked. Liver regeneration has more complex mechanisms, such as the transdifferentiation between cell types or hepatic progenitor cells mediated. Dysregulation of liver regeneration causes severe chronic liver disease. Gaining a more comprehensive understanding of liver regeneration mechanisms would facilitate the advancement of efficient therapeutic approaches. This review provides an overview of the signalling pathways linked to different aspects of liver regeneration in various liver diseases. Moreover, new knowledge on cellular interactions during the regenerative process is also presented. Finally, this paper explores the potential applications of new technologies, such as nanotechnology, stem cell transplantation and organoids, in liver regeneration after injury, offering fresh perspectives on treating liver disease.

## Graphical abstract

- This article summarises the signalling pathways associated with various aspects of liver regeneration in different types of liver injury.
- This article introduces new knowledge of cellular interactions during regeneration.
- This article explores the potential application of new technologies such as nanotechnology, stem cell transplantation and organ tissues in liver regeneration after injury.

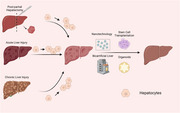

## 1 INTRODUCTION

**FIGURE 1:**
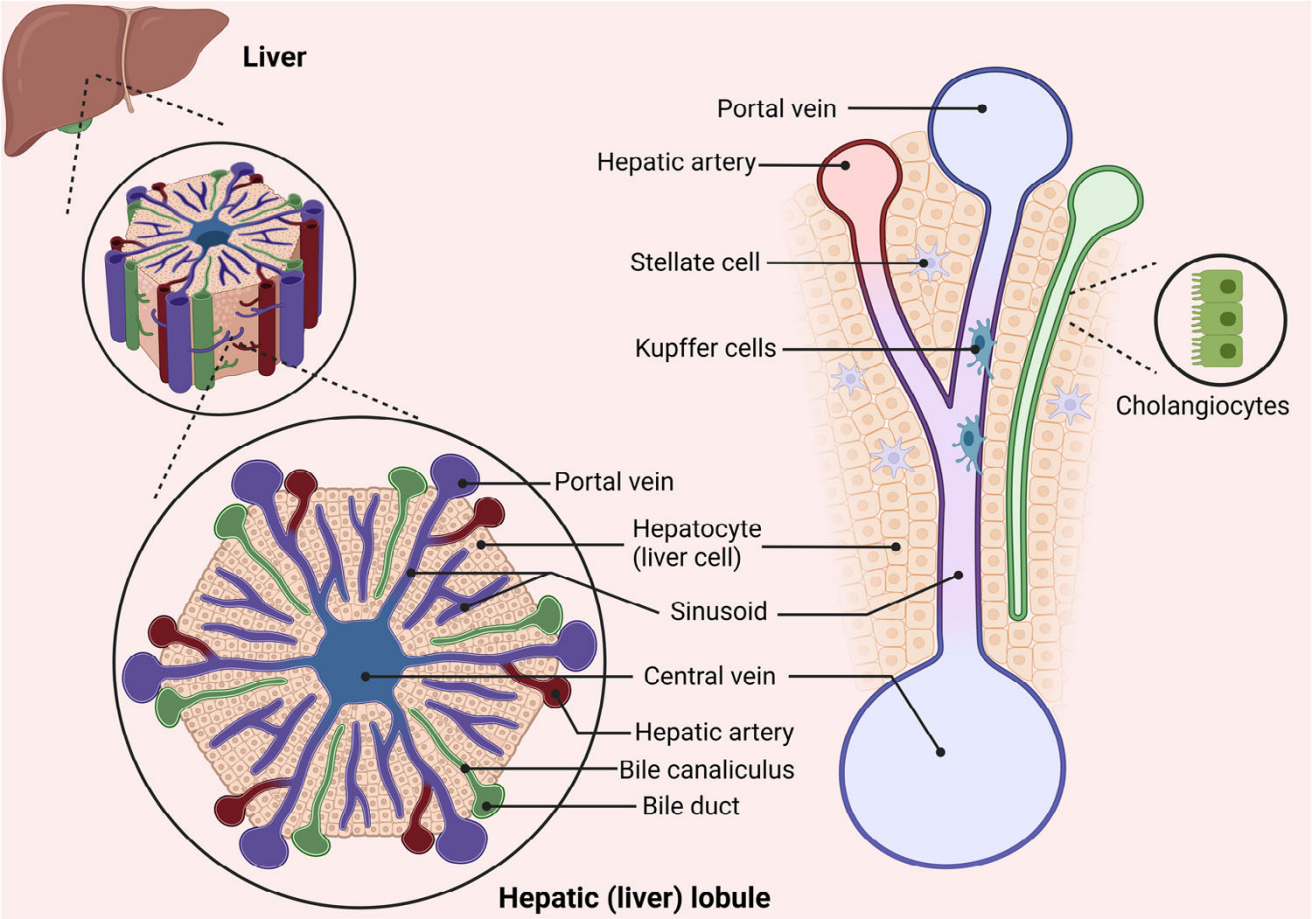
Schematic distribution of various cells in the liver lobules.

The liver has a complex structure and performs various functions such as metabolism, detoxification, immunity, haematopoiesis, blood storage, blood volume regulation and coagulation. The liver parenchyma comprises two kinds of epithelium: hepatocytes and cholangiocytes. The non‐parenchymal section consists of liver sinusoidal endothelial cells (LSECs), hepatic stellate cells (HSCs), Kupffer cells (KCs), smooth muscle cells, fibroblasts and different immune cells.^1^ Scientific evidence has demonstrated that the regenerating ability of the adult liver is nearly equivalent to that of a foetal liver.^2^, ^3^ When observed in two dimensions, the hepatic lobules create a hexagonal pattern with six inlet areas encircling the central vein (CV).^4^ The liver lobule is anatomically separated into three distinct regions: Zone 1 located near the junction of the portal vein; Zone 2 situated in the intermediate region; and Zone 3 surrounding the CV.^5^ Emerging studies indicated that intricately cooperation between hepatocytes and surrounding NPCs are crucial for liver regeneration (Figure 1).

In normal livers, 99% of hepatocytes are in the quiescent (G0) phase, and DNA labelling studies reveal that less than 0.2% of hepatocytes engage in DNA synthesis. Mouse hepatocytes have a life cycle of approximately 200–300 days.^3^ Under typical physiological circumstances, the replication of hepatocytes is able to sustain the normal functioning of the liver. Upon acute liver injury, the major regeneration mechanism is the compensatory replication of surviving hepatocytes. However, in cases of severe chronic liver disease (CLD), when massive hepatocytes were lost and hepatocyte replication were arrested, the liver will take advantages of other ways, for example, regeneration by liver progenitor cells (LPCs).^6^, ^7^ In the past decades, a great deal of study has been done on the basic mechanism of liver regeneration. Hepatocytes are the principal cells responsible for the liver's physiological activities, and their origin is the core of liver regeneration research. The plasticity between cholangiocytes and hepatocytes, as well as their roles as sources of new hepatocytes after injury, have attracted much attention.^8^ It has been found that in addition to parenchymal cells, non‐parenchymal cells (NPCs), such as HSCs and LSECs, also have crucial functions in regulating liver regeneration.^9^ Moreover, the applications of nanotechnology, stem cell therapy organoids and other emerging technologies provide new research directions and treatment possibilities for liver regeneration.^10^

In this review, this study provides a concise summary of the most recent research discoveries about liver regeneration in various liver diseases, while also examining the current obstacles faced in this field. The latest interventions associated with liver regeneration and treatment prospects for liver disease were also described. Through these comprehensive studies, this paper hopes to provide new insights into the mechanism of disease‐related liver regeneration and provide some ideas for future clinical applications.

## 2 DIFFERENT RESEARCH MODELS FOR LIVER REGENERATION

**FIGURE 2:**
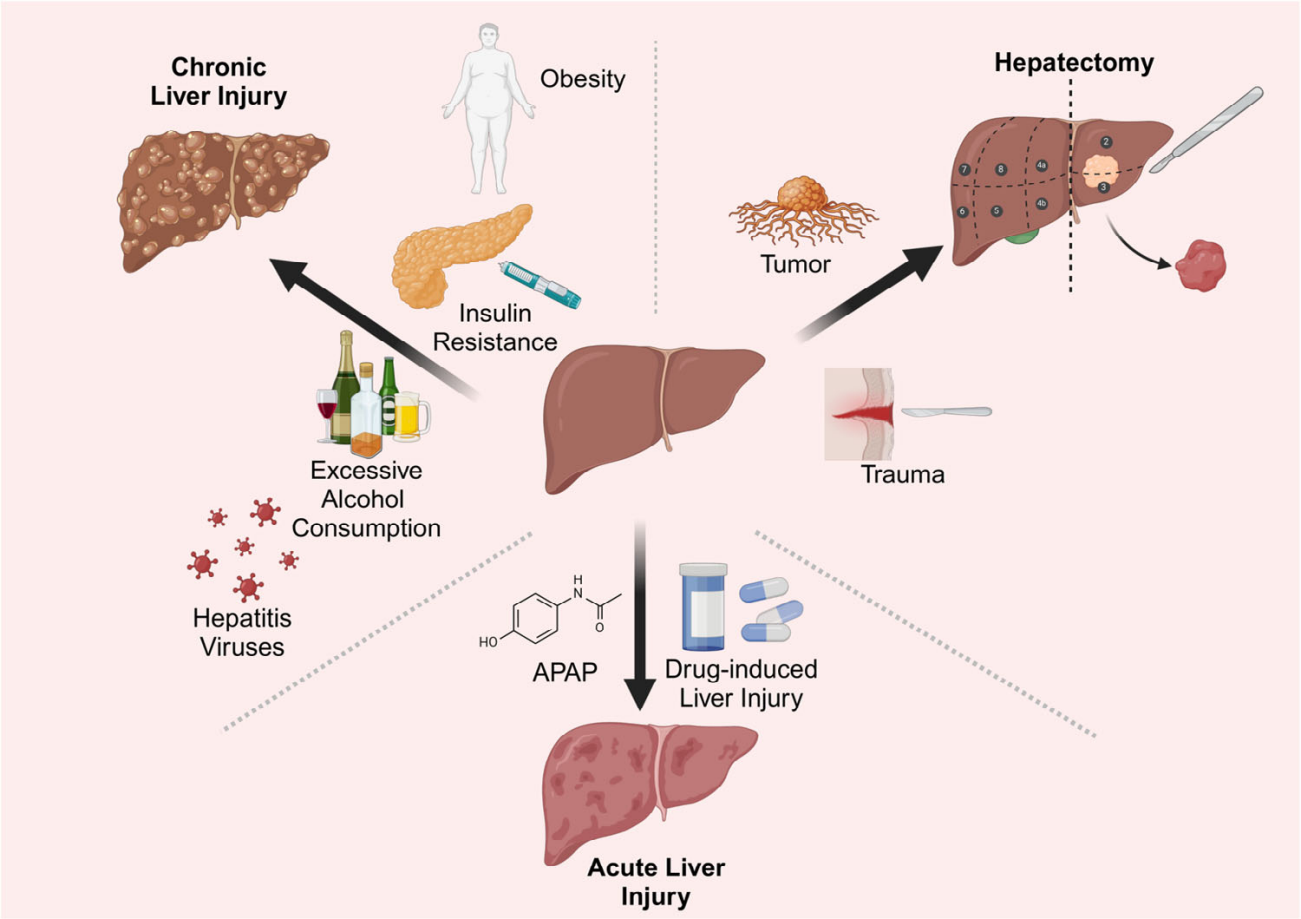
Various factors leading to liver injury.

Various models have been used in liver regeneration studies to induce liver injury and subsequent regeneration. Liver regeneration models are categorised into two groups: the post‐partial hepatectomy (PHx) models and chemically induced liver injury models.^11^, ^12^ These models help researchers understand how the liver responds and regenerates in the context of different types of injury and liver disease (Figure 2).

### 2.1 The post‐PHx models

In PHx models, a surgical procedure is performed to remove a specific section of the liver without causing direct harm to liver cells. This model mainly accomplishes liver regeneration by activating the proliferation of residual liver cells.^12^, ^13^ Liver regeneration models of PHx employ the multi‐lobular structure of the rodent liver. This involves surgically removing certain lobes without inducing necrosis in the remaining lobes. In rat PHx model, approximately 66% of the liver mass will be excised, whereas in the mouse PHx model, around 50% is taken out.^14^, ^15^ The closest human equivalent to this model is PHx that most commonly performed for the resection of primary or secondary liver tumours.^16^ This model is primarily applied to regenerative capacity study and is able to reliably mimic the regenerative process after human liver resection and is performed in the absence of chemical damage to the liver. In this model, the residual hepatic lobe enlarges to achieve the liver mass prior to PHx within about a week.^14^ During regeneration, the initial response is hepatocyte hypertrophy, followed by proliferation or hyperplasia of non‐epithelial components.^17^ Following liver regeneration, the lobules and bile ducts enlarge, with the hepatocyte plate width usually increasing from 1 to 1.5 hepatocytes, bordered by hepatic sinuses on each side.^6^ Multiple studies suggest that in PHx‐induced liver regeneration cells such as hepatocytes and cholangiocytes generate new cells of the same kind, characterised by phenotypic fidelity.^3^, ^18^ This phenomenon contrasts with the cellular plasticity discussed later. Liver regeneration following PHx also occurs in humans. For instance, liver volume regeneration is most pivotal in the first 2 weeks following living donor transplantation, nearing maximum within 2 months.^16^, ^19^ The liver's high regenerative capacity makes the model very valuable for researching fundamental biological processes and regenerative mechanisms. However, the model is more invasive and requires higher surgical skills. Nevertheless, it is incapable of replicating liver damage induced by specific causes, such as viral infection or exposure to chemical toxins.^6^

### 2.2 Chemically induced liver injury models

Chemically induced liver injury models, such as the use of diethylnitrosamine, carbon tetrachloride (CCl_4_) or ethanol, can directly damage liver cells, triggering acute or chronic liver damage.^20^, ^21^ These models can more accurately replicate the conditions seen in human liver diseases like hepatitis, liver fibrosis and cirrhosis, making them commonly utilised in drug development and research on treatments for liver disease.

#### 2.2.1 Acute liver injury models

Acute liver injury often occurs when a high dosage of a toxin is given in a single instance. This model is employed to investigate how the liver reacts to acute injury and how it recovers.^22^, ^23^ Acute liver injuries are usually caused by short‐term, intense factors such as exposure to large doses of chemical toxins. After acute damage to the liver, the remaining liver cells rapidly begin to replicate.^24^ Liver regeneration mainly relies on the proliferation of relatively healthy residual hepatocytes.^11^, ^25^ It can simulate the common clinical situation of drug‐induced liver injury. It is highly relevant for studying the immediate response and regenerative processes after liver injury. Acute models may not be able to cover the extracellular matrix (ECM) alterations and inflammatory responses that occur during chronic lesions. The impact on long‐term liver function is more difficult to assess.

#### 2.2.2 Chronic liver injury models

Different from acute injury‐induced regenerative repair, chronic injury induced by sustained mild or moderate‐intensity injury factors, for instance, long‐term excess alcohol consumption or chronic viral hepatitis. Chronic liver injury models mimic chronic liver conditions by administering small amounts of chemical triggers like CCl_4_ or inducing fatty liver through diet for an extended duration (typically spanning weeks to months).^26^ This simulation replicates the development of long‐term liver conditions like liver fibrosis and cirrhosis, making it ideal for researching persistent inflammation, immune reactions and their impact on liver recovery. It requires a longer period of time before pathological changes can be observed. Also, the model is more demanding on animals and more costly to maintain.^26^, ^27^ The liver regeneration process is more complex and involves more cell types.^28^, ^29^ However, long‐period administration of chemoinducers may lead to serious side effects.^30^ The mechanism of injury of some chemicals may not be fully consistent with the specific mechanism of human diseases.

## 3 MECHANISMS UNDERLYING LIVER REGENERATION

### 3.1 Liver regeneration after resection

The liver regeneration procedure after PHx entails the intricate interplay of multiple elements and mechanisms to guarantee the restoration of the liver's functional mass. This process is not only essential for patients undergoing liver resection due to tumours or other diseases but also provides valuable insights into the liver's remarkable regenerative capabilities. Knowledge of the critical factors impacting liver regeneration can help in creating treatment plans to boost recovery and enhance patient results. Many factors influence liver regeneration after resection, such as cytokines, growth factors and bile acids.

#### 3.1.1 Growth factors influencing regeneration after liver resection: hepatocyte growth factor and epidermal growth factor

Restoring complete blood flow is crucial for liver regeneration.^16^ Important molecules that are activated upon the prompt restoration of blood flow are hepatocyte growth factor (HGF) and epidermal growth factor (EGF).^31^ HGF is secreted by hepatic macrophages and mediates hepatocyte proliferation through its receptor c‐Met, while activating downstream effectors such as extracellular signal‐regulated kinase (Erk1/2), protein kinase B (AKT) and signal transducer and activator of transcription 3 (STAT3), driving hepatocyte proliferation and survival.^32^, ^33^ EGF, through binding its receptor EGFR, initiates the hepatocyte proliferation signalling pathway, promoting hepatocyte proliferation and survival.^34^ In the PHx model, after blood flow was restored, HGF and EGF were rapidly activated to promote hepatocyte division and proliferation.

**FIGURE 3:**
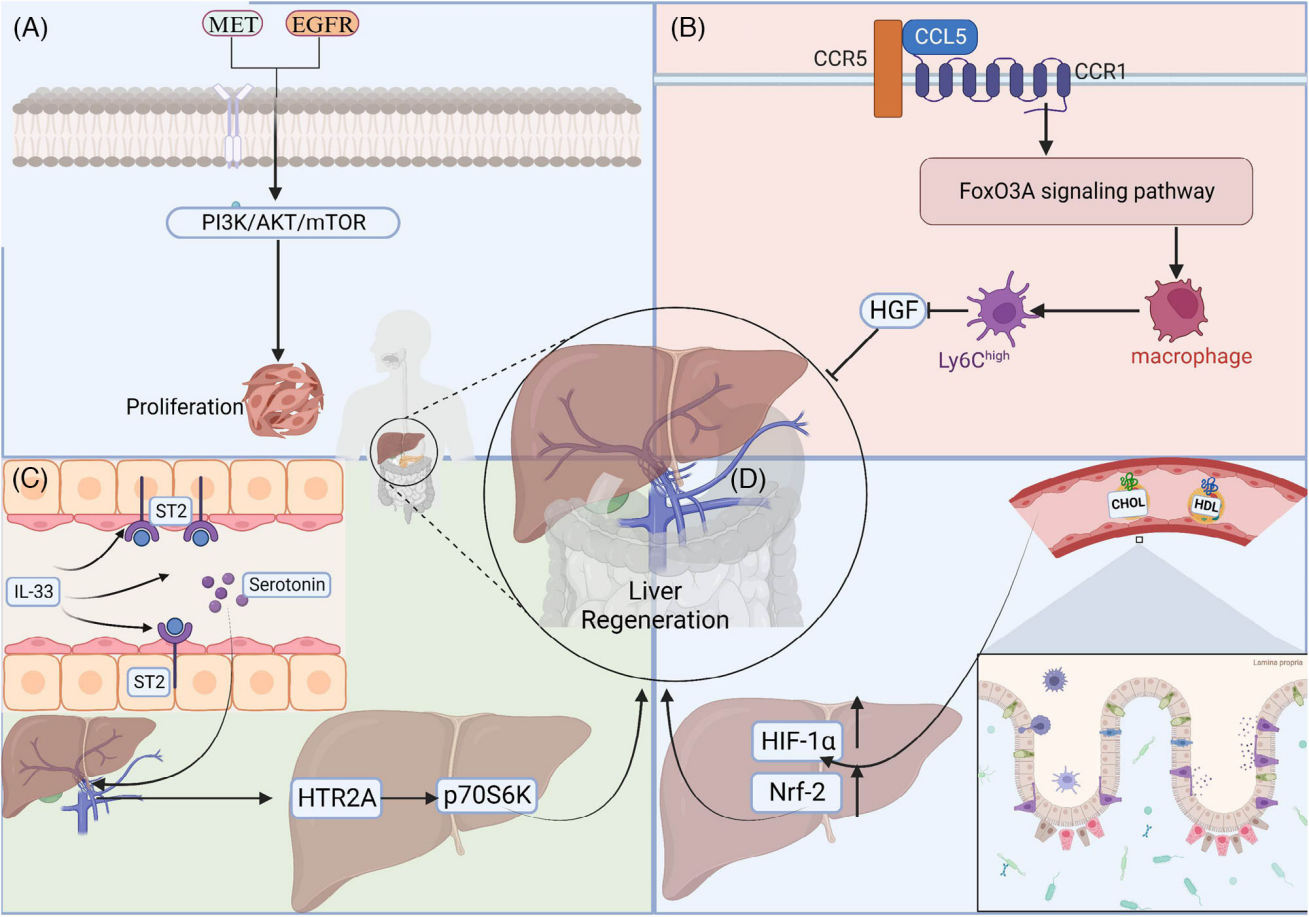
Signalling pathways for liver regeneration after resection. (A) Activation of MET and EGFR‐related signalling pathway activation can cause hepatocyte proliferation. (B) CCL5 binds via CCR1 and CCR5 and activates the Fox03a pathway to induce a proinflammatory Ly6C(hi) phenotype in macrophages, thereby inhibiting HGF production and delaying regenerative recovery. (C) IL‐33 binds to its receptor ST2 and induces intestinal mucosal cells to release more serotonin into the portal blood stream, activating HTR2A/p70S6K13 in hepatocytes to promote liver regeneration. (D) Cholesterol supplementation stimulates HIF‐1α and Nrf‐2 gene expression and induces hepatic inflammation and hepatocyte proliferation.

The signalling pathways of EGFR and MET are essential for liver regeneration and healthy hepatocyte function. Mice with abnormal MET and EGFR signallings displayed lower liver‐to‐body ratios, worse hepatic metabolism and higher rates of cell death. This disruption eliminates liver regeneration post‐hepatectomy, leading to liver failure and, occasionally, death within 12–14 days after liver resection^32^ (Figure 3). Pre‐treating diet‐induced obese mice with meloxicam dramatically enhances the expression of EGFR protein in hepatocytes. Following hepatectomy, meloxicam administration improved liver damage, enhanced hepatocyte division and promoted liver mass regeneration in overweight mice by 70%. Meloxicam treatment post‐hepatectomy improved the survival rate of mice by 80%.^35^ HRX215, a small molecule inhibitor of MKK4 kinase, has been found to effectively promote liver regeneration.^36^ In a study using a pig liver resection model, HRX215 demonstrated significant efficacy: 85% of the treated subjects, regardless of whether the inhibitor was administered before or after surgery, did not exhibit typical signs of liver failure. The treatment group showed higher survival rates and healthier liver status compared with the control group.^36^

#### 3.1.2 WNT proteins and signals

The liver regeneration process after hepatic injury relies heavily on the Wnt/β‐catenin signalling. Following PHx, the Wnt/β‐catenin axis is activated to facilitate quick liver regeneration through stimulating the proliferation and differentiation of hepatocytes. Studies indicate that increased stability and activity of β‐catenin directly facilitate hepatocytes enter G1 phase, promoting DNA synthesis and cell division^37^, ^38^ Additionally, by controlling downstream effector target genes such as cyclin D1 and c‐Myc, Wnt signalling promotes hepatocyte proliferation and tissue repair.^39^, ^40^ Post‐hepatectomy, Wnt signalling interacts with other signalling pathways like Notch and HGF, forming a complex regulatory network that collectively drives liver regeneration.^41^, ^42^

#### 3.1.3 Cytokines

Interleukins like IL‐6 and IL33 exert important roles in liver regeneration.^31^ After hepatic resection and acute liver injury, IL‐6 contributes a lot to liver regeneration.^43^ IL‐6, mainly secreted by KCs, activates the Janus Kinase (JAK)–Signal Transducer and Activator of Transcription 3 (STAT3) signalling pathway after binding to its receptor, IL‐6R. IL‐6–JAK–STAT3 signalling promotes the G1/S transition of hepatocytes via up‐regulating cyclinD1expression.^43^, ^44^ In addition, IL‐6 also enhances hepatocyte survival through the STAT3 signalling pathway.^45^, ^46^ STAT3 activation induces the up‐regulation of anti‐apoptotic genes like Mcl‐1 and Bcl‐2, thereby enhancing the anti‐apoptotic ability of hepatocytes in the injury environment.^46^, ^47^ Furthermore, IL‐6 plays an important immunoregulation role during liver regeneration. IL‐6 regulates the development and function of T cells and B cells, maintains a balanced inflammatory response and protects hepatocytes from excessive inflammation‐induced damage.^48^, ^49^ Meanwhile, IL‐6 promotes the construction of a regenerative environment by regulating the polarisation state of macrophages, which is conducive to the repair and regeneration of hepatocytes.^49^, ^50^ Patients having hepatectomy and mice after PHx exhibited increased IL‐33 levels. IL‐33 promotes regeneration in hepatic resection models, and the deficiency of IL‐33 or its receptor ST2 leads to tumour growth inhibition and liver regeneration delay. IL‐33 induces intestinal mucosal cells to release more serotonin into the portal bloodstream, activating HTR2A/p70S6K in hepatocytes.^51^

The chemokine C‐C motif chemokine ligand 5 (CCL5) stimulates the proinflammatory development of macrophage through the forkhead box O 3a pathway, which is regulated by CCR1 and CCR5. This process inhibits the generation of HGF and delays regenerative recovery.^52^ Additional cytokines such as Tumor Necrosis Factor (TNF)‐α and transforming growth factor (TGF)‐α also have a distinctive influence in controlling the growth and survival of hepatocytes through specific signalling pathways.^53^

#### 3.1.4 Bile acids

Hepatocytes create bile acids, which are then discharged into the biliary duct and intestines before being absorbed again. Subsequently, bile acids returned to the liver via the enterohepatic circulation. Bile acid levels dramatically increased immediately after PH in rodents and humans.^54^, ^55^ Bile acids stimulate the farnesoid X receptor (FXR) and G‐protein‐coupled bile acid receptor 1 (GPBAR1, also referred to as TGR5), playing essential functions in regulating bile acid balance and preventing liver damage. FXR activation stimulates the expression of genes involved in bile acid synthesis, detoxification and transport. This helps to reduce liver cell damage caused by bile acids and supports the regeneration of the liver. Furthermore, bile acids modulate the activity of various growth and regeneration‐related pathways, for instance, the Wnt/β‐catenin signalling. Moreover, FXR and TGR5 signalling have been demonstrated not only to promote hepatocytes proliferation but also to protect hepatocytes from apoptosis, thereby facilitating liver regeneration following injury or PHx.^56^, ^57^

### 3.2 Acute liver injury and liver regeneration

Various factors can lead to acute liver injury in clinical settings, including acetaminophen poisoning, alcoholic hepatitis, virus infections like hepatitis A, B and E and autoimmune hepatitis. APAP poisoning is the leading cause of acute liver failure.^58^ Here, we primarily focus on APAP‐induced acute liver injury and regeneration.

#### 3.2.1 Mechanisms of APAP‐induced liver injury

APAP is safe at normal doses, but excessive APAP will result in the accumulation of the toxic metabolite N‐acetylresorcinol (NAPQI), leading to hepatotoxicity. At normal doses, APAP is metabolised mainly by the hepatic sulphate and glucuronosyltransferase systems.^59^ Only a small portion is oxidised to form NAPQI via cytochrome P450 enzymes (mainly CYP2E1). When there is too much NAPQI produced in the liver, it surpasses the detoxification ability of glutathione, resulting in the formation of covalent bonds with intracellular lipids and proteins. This leads to cellular damage and death. The buildup of NAPQI causes oxidative stress, DNA damage and disruption of cell signalling pathways, ultimately leading to hepatocyte necrosis.^60^, ^61^

Excessive APAP exposure damages mitochondria and results in significant DNA harm, resulting in swift cell death during specific phases of the cell cycle and potentially halting the entire process. Alterations in the activity of lower‐level detectors and controllers of DNA damage lead to halting of cell cycle progression at the G1/S checkpoint, postponement of S phase entry and G2 progression. Individuals experiencing sudden liver failure display DNA harm in the liver and abnormalities related to cell division, such as hepatocytes being limited to an abnormal number of chromosomes. However, treating cells with protective cytokines can reverse the APAP‐induced cell cycle restriction and restore full circulation.^62^

#### 3.2.2 Role of P53 in APAP‐induced liver injury

P53 has multiple functions in the pathogenesis of acute liver damage induced by APAP poisoning: it prevents further liver damage by maintaining metabolic balance and regulating liver regeneration initiation through proliferative signalling. Experiments revealed that liver regeneration initiation was notably delayed in p53 knockout mice, but once initiated, the cell cycle proceeded much faster than in wild mice due to sustained signalling.^63^ Moreover, p53 is essential in regulating the response to oxidative stress in APAP‐induced liver damage. During an APAP overdose, p53 is stabilised due to the inhibition of its sulfation by PAPSS2, leading to enhanced p53‐p21‐Nrf2 signalling. This pathway significantly enhances the liver's antioxidative capacity, leading to alleviate liver damage and increased survival rates of mice. The inhibition of p53 sulfation disrupts its interaction with MDM2, preventing p53 ubiquitination and degradation, which further augments its protective effects against APAP‐induced oxidative stress. Targeting p53 sulfation as a therapeutic strategy for APAP‐induced acute liver failure is emphasised by this mechanism.^64^

#### 3.2.3 Role of CYP2E1 in APAP‐induced liver injury

**FIGURE 4:**
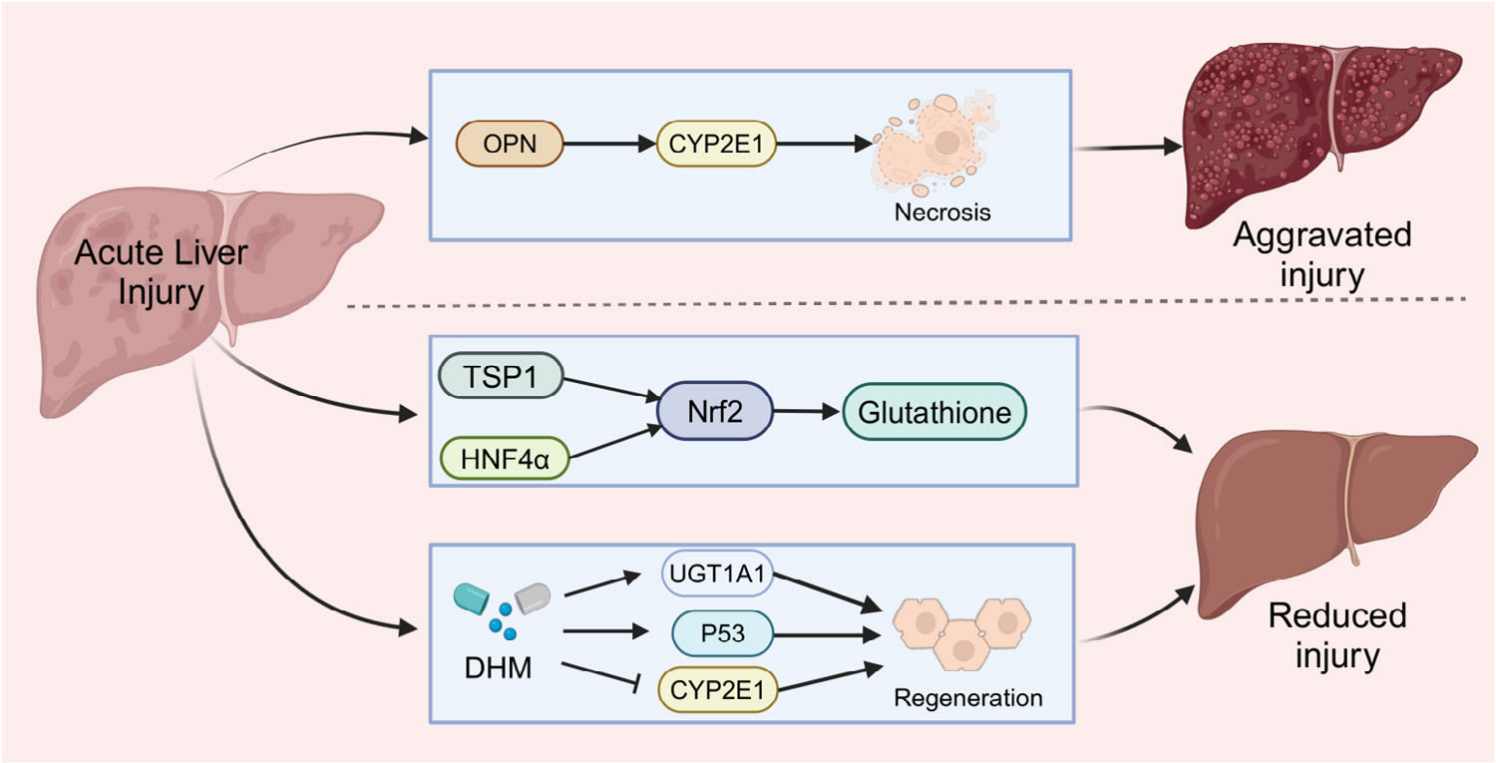
Some pathways activated in acute liver injury. OPN exacerbates APAP‐induced liver injury by increasing CYP2E1 secretion. TSP‐1 and HNF‐4α acted with Nrf2 to increase glutathione levels and attenuate APAP‐induced liver injury, respectively. DHM activated UGT1A1 and P53 to promote the associated regeneration and inhibited CYP2E1 expression to attenuate APAP‐induced liver injury.

Suppression of cytochrome P450 family 2 subfamily E member 1 (CYP2E1) expression impairs the APAP metabolism. Dihydromyricetin (DHM) mitigates APAP‐induced liver injury via regulating proteins that are related to cell death and liver regeneration, including activating UDP‐glucuronosyltransferase 1A1 (UGT1A1), promoting p53‐associated regeneration and suppressing CYP2E1 expression.^65^ Furthermore, in a mouse model of hepatotoxicity generated by an overdose of APAP, both serum and liver levels of osteopontin (OPN) showed a considerable rise. OPN protein secretion primarily originates from dying or dead hepatocytes. OPN worsens APAP‐induced liver damage by speeding up the breakdown of APAP through increased production of CYP2E1. In addition, while OPN deficiency initially protected against APAP‐induced liver damage, it ultimately delayed the healing process by making hepatocytes more susceptible to cell death and hindering liver regeneration^66^ (Figure 4).

#### 3.2.4 The role of glutathione in APAP‐induced liver injury

Multiple researches have proved that adding glutathione is essential for the regeneration and healing of the liver after an overdose of APAP, in which Nrf2 is a critical factor. Thrombospondin‐1 (TSP‐1), a homotrimeric protein, interacts with proteins like Nrf2 to initiate antioxidant signalling. Additionally, TSP‐1 can activate TGF‐β1, potentially worsening liver injury. In APAP‐treated mice, knocking down the TSP‐1 protein decreases TGF‐β1 signalling, but results in more liver damage and increased cell death because of lower Nrf2 expression and glutathione activity.^58^ Hepatocyte nuclear factor 4 alpha (HNF‐4α) works with Nrf2 to boost glutathione levels, helping in the healing of liver damage caused by APAP, a process blocked by cMyc^67^ (Figure 4 and Table 1).

**TABLE 1:** Key molecules and their mechanisms in APAP‐induced liver injury.

Relevant factors	Mechanism of action	Impact and consequences	References
p53	Regulates metabolic balance, liver regeneration initiation, modulates oxidative stress response; stabilised by inhibition of sulfation leading to enhanced signalling and reduced degradation	Delays liver regeneration in knockout mice, enhances antioxidative capacity, improves survival rates, potential therapeutic target for acute liver failure	^63^, ^64^
CYP2E1	Metabolises APAP; its expression is suppressed by dihydromyricetin, which also activates liver regeneration pathways	Reducing CYP2E1 expression mitigates liver damage, but increased expression through OPN exacerbates damage	^65^, ^66^
OPN (osteopontin)	Secreted by dying hepatocytes, increases CYP2E1 expression, accelerates APAP metabolism	Initially protects against hepatotoxicity, but later sensitises hepatocytes to apoptosis and impairs liver regeneration	^66^
Glutathione	Supplementation supports liver regeneration; interacts with proteins like Nrf2 to enhance antioxidant responses	Crucial for recovery post‐overdose, modulation by other proteins (TSP‐1, HNF‐4α) influences liver injury outcomes	^66^, ^67^
ANXA2^+^ hepatocytes	Facilitates necrotic wound closure, enhances HGF‐induced migration and cytoskeletal reorganisation	Critical in liver regeneration; absence reduces migration but not proliferation, interacts with Wnt/β‐catenin pathway for liver recovery	^3^, ^68^

#### 3.2.5 Discoveries at the single‐cell level

The ANXA2^+^ migratory hepatocyte subpopulation is essential for liver regeneration, primarily by facilitating necrotic wound closure through collective migration.^68^ The presence of ANXA2^+^ hepatocyte enhances HGF‐induced hepatocyte migration and shows significant cytoskeletal reorganisation during migration.^68^ HGF signalling promotes cytoskeletal protein reorganisation, thereby enhancing cell migration capability. The absence of ANXA2^+^ hepatocyte reduces HGF‐induced migration but does not affect hepatocyte proliferation. The Wnt/β‐catenin axis improves liver function and promotes liver regeneration by controlling the activity of ANXA2^+^ hepatocytes.^3^, ^68^

### 3.3 CLD and liver regeneration

#### 3.3.1 Mechanisms underlying liver injury in the context of CLD

CLD arises from various sources such as non‐alcoholic fatty liver disease (NAFLD), excessive alcohol consumption or viral hepatitis. Alcoholic hepatitis is a condition where the liver becomes inflamed due to long‐term and excessive alcohol usage. It is a type of liver disease related to alcohol abuse that can lead to the development of liver fibrosis or cirrhosis.^69^ Ethanol is first metabolised to acetaldehyde in the liver by enzymes, the main enzymes involved being alcohol dehydrogenase and CYP2E1. Acetaldehyde is further metabolised to acetic acid by the enzyme acetaldehyde dehydrogenase.^70^, ^71^ Acetaldehyde is a highly reactive compound that can cause protein and DNA damage, increase oxidative stress and stimulate inflammatory responses.^71^ Long‐term drinking of alcohol can result in fat buildup in liver cells, triggering KCs and other immune cells to release inflammatory substances that may lead to additional liver cell harm and fibrosis.^72^ Both mechanisms of liver injury involve not only metabolic abnormalities and oxidative stress but also extensive alterations in intracellular signalling pathways.

NAFLD, a common cause of CLD, is mainly linked to metabolic syndrome, obesity, insulin resistance and dyslipidaemia. NAFLD includes a range of liver disorders that go from basic steatosis (accumulation of fat in the liver) to non‐alcoholic steatohepatitis (NASH), which may advance to fibrosis, cirrhosis and hepatocellular carcinoma.^73^ The development of NAFLD is caused by various factors occurring simultaneously, such as insulin resistance causing an increase in free fatty acid flow to the liver, oxidative stress, lipid peroxidation and dysfunction of mitochondria.^74^

Viral‐induced hepatitis, specifically infections caused by hepatitis B virus (HBV) and hepatitis C virus (HCV), are additional major contributors to CLD. HBV and HCV result in persistent liver inflammation, which can ultimately result in the development of fibrosis, cirrhosis and hepatocellular cancer. HBV inserts itself into the host genome and can evade immune surveillance, leading to persistent infection.^75^ HCV, an RNA virus, induces chronic inflammation through continuous replication and production of viral proteins that trigger immune responses and liver cell injury.^76^

Regardless of its aetiology, CLD often leads to prolonged hepatocyte loss, resulting in liver fibrosis, cirrhosis and liver tumours.^77^ Following major hepatic resection or acute liver injury, liver regeneration primarily occurs in relatively healthy remnant hepatocytes, a process often referred to as physiological regeneration. In these situations, mature hepatocytes restore liver function through proliferation. Although LPCs play a lesser role in these cases, they may be activated and participate in regeneration when hepatocytes have limited proliferative capacity or are excessively injured.

In CLD, liver regeneration is more complicated. LPCs‐mediated regeneration is particularly pronounced in these contexts and is usually accompanied by the transdifferentiation of hepatocytes and cholangiocytes, a phenomenon referred to as pathological regeneration. Overall, the main feature of regeneration in chronically injured livers is the coexistence of hepatocyte self‐replication and regeneration mediated by LPCs or other cells. The dominance of particular mechanisms relies on the extent of hepatocyte proliferative capacity impairment and the seriousness of the injury. During the initial phases of many chronic liver conditions, hepatocyte self‐replication may still predominate, but LPCs or other cell‐mediated regeneration may become more important as the disease progresses and the injury worsens.

#### 3.3.2 Liver regeneration originated from biliary epithelial cells

Recent studies have elucidated the complexity of liver regeneration in chronic disease.^78^, ^79^, ^80^ For instance, research has highlighted the role of transitional LPCs (TLPCs) in situations when hepatocyte‐mediated regeneration is compromised. TLPCs originate from biliary epithelial cells (BECs) and possess the ability to differentiate into hepatocytes.^80^ A dual genetic lineage tracing method was used in the research to mark TLPCs and monitor their differentiation trajectory. TLPCs were discovered to have the ability to differentiate into hepatocytes or revert back to a BECs fate, demonstrating their bipotency. This plasticity is essential for liver regeneration under conditions where hepatocyte proliferation is limited. Mechanistically, the study showed that Notch signalling is pivotal in maintaining the BECs identity and preventing their transition to TLPCs.^80^ On the other hand, the Wnt/β‐catenin pathway encourages TLPCs to develop into fully functional liver cells. The inhibition of Notch signalling in BECs was shown to enhance the activation of TLPCs, increasing their conversion to hepatocytes.^80^

Researchers used snRNA‐seq and advanced 3D imaging on liver biopsies from patients at different MASLD stages to uncover notable alterations in liver structure and cell activity. They discovered that hepatocytes lose their zonation and that biliary tree undergoes considerable reorganisation.^78^ They discovered that hepatocytes lose their zonation and that biliary tree undergoes considerable reorganisation. Crucially, the conversion of hepatocytes to cholangiocytes happens without the involvement of adult stem cells or developmental progenitors.^78^ Cholangiocyte organoids demonstrated the importance of the PI3K–AKT–mTOR pathway in functional validations, connecting it to insulin signalling.^78^, ^79^ This pathway, along with others such as Wnt/β‐catenin and TGFβ signalling, orchestrates the cellular plasticity necessary for liver regeneration in the face of chronic injury.

#### 3.3.3 Liver regeneration originated from LPCs

LPCs are oval‐shaped liver resident stem cells located in Hering's duct at the junction of liver parenchymal cells and the confluence area, resembling biliary BECs in morphology and volume. LPCs show the presence of stem cell indicators like EPCAM, Sca‐1, CD133, CD24, A6, Trop2 and Lgr5, in addition to cholangiocyte markers SOX, CK19 and hepatocyte markers HNF‐4α, CK8. LPCs are bipotent stem/progenitor cells that are capable to differentiate into both hepatocytes and cholangiocytes.^24^ LPCs also have other sources, for example, in some circumstances, mature hepatocytes or cholangiocytes can act as parthenogenetic stem cells, transforming into each other to restore normal liver architecture architecture.^3^

Hepatocytes, which are fully developed liver cells, can undergo a process called ductal metaplasia under chronic injury, where they transform into hepatic progenitor cells. This transformation is reversible and allows the cells to avoid further damage. Once the injury stops, these hepatic progenitor cells can then differentiate back into fully functional hepatocytes.^81^ CD24^+^LCN2^+^ LPCs are mainly derived from non‐substantial cells of the liver, such as BECs and existing LPCs. Recently, it has also been demonstrated that hepatic progenitors of non‐hepatocyte origin make a very limited contribution to the regeneration of the mouse liver.^82^

In vitro, the transformation of hepatocytes and hepatic progenitor cells can be induced by replicating a human environment that is conducive to liver regeneration. The in vivo environment supporting liver regeneration was mimicked using specific small molecules and growth factors, such as HGF in combination with Y‐27632, A‐83‐01 and CHIR99021, which resulted in the in vitro conversion of mature hepatocytes into proliferative‐expandable hepatic progenitor cells.^81^, ^83^, ^84^ Meanwhile, co‐culturing human LPCs (HepLPCs) with human umbilical vein endothelial cells (HUVECs) effectively mimicked the in vivo microenvironment of the human liver, and the glial cell‐derived neurotrophic factor secreted by the HUVECs facilitated the transformation of HepLPCs into mature hepatocytes through activation of the Met signalling pathway.^85^

#### 3.3.4 Signalling pathways for the LPCs differentiation

Extensive studies have been conducted on signalling pathways that regulate LPCs differentiation, including Wnt/β‐catenin, Notch, HGF/c‐Met and Hippo/YAP signalling. Wnt/β‐catenin signalling is associated with LPCs differentiation into hepatocytes. In rats treated with 2‐ethoxytoluamide and major hepatic resection, β‐catenin activity significantly increased during LPCs proliferation. Conversely, LPCs numbers decreased substantially without β‐catenin, indicating its key role in LPCs activation and proliferation.^34^

**FIGURE 5:**
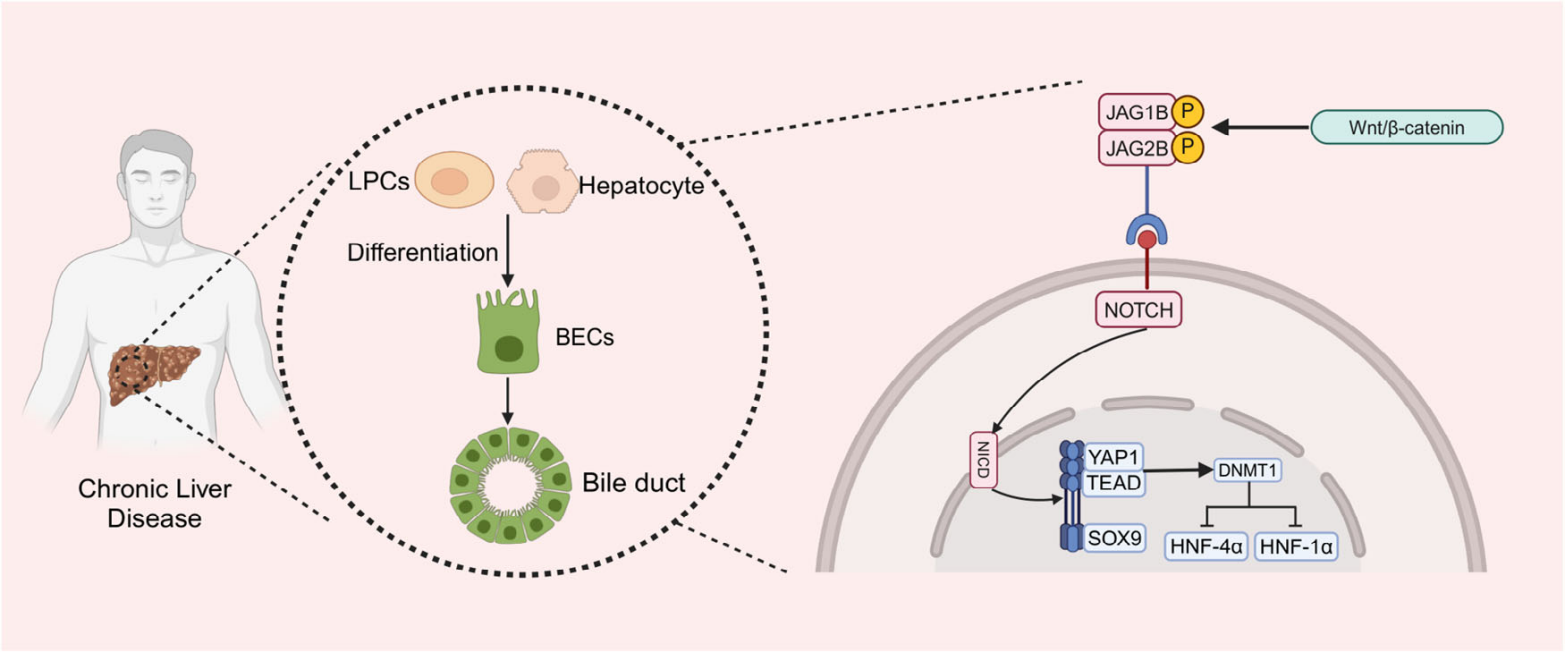
NOTCH–YAP1/TEAD–DNMT1 axis is critical for the differentiation of LPCs/hepatocytes into BECs. When Wnt/β‐catenin signalling is enhanced, the levels of Notch ligand genes JAG1B and JAG2B are elevated in hepatocytes. Afterwards NICD can regulate downstream SOX9 and YAP1. DHMT1, a downstream effector of the YAP1–TEAD complex, directs the conversion of LPCs/hepatocytes to BECs by repressing hepatocyte‐specific genes (e.g., HNF‐4α, HNF‐1α).

It has been demonstrated that the phagocytosis of hepatocyte debris by macrophage cells induces Wnt3a expression and activation of classic Wnt/β‐catenin signalling in neighbouring LPCs, leading to differentiation into hepatocytes.^86^ Research have shown that interfering with or excessively stimulating Wnt/β‐catenin signalling can hinder the formation of the biliary tract within the liver of zebrafish. Suppression of the Wnt/β‐catenin pathway leads to decreased Notch function in BECs. Inhibiting Wnt/β‐catenin signalling in hepatocytes reduces Notch activity in BECs. The levels of JAG1B and JAG2B Notch ligand genes decrease in liver cells when Wnt/β‐catenin signalling is blocked, and rise in liver cells when Wnt/β‐catenin signalling is heightened. These findings indicate that the liver's Notch activity is controlled by Wnt/β‐catenin signalling. Crucially, the restoration of Notch activity can effectively fix the damage to the biliary system produced by the suppression of Wnt/β‐catenin^87^ (Figure 5).

The Notch pathway is a highly conserved controller of cell growth and the upkeep of stem/progenitor cells in different tissues. It has been demonstrated to have significant functions in the differentiation of bile ducts. Alagille syndrome, caused by congenital Notch deficiency, results in biliary defects and subsequent cholestasis. For instance, Alagille syndrome, which is caused by a congenital deficiency in Notch signalling, leads to biliary defects and cholestasis.^88^ During biliary regeneration, myofibroblasts express Jagged1, promoting Notch signalling in LPCs and facilitating their differentiation into cholangiocytes.^89^ EpCAM controls the transformation of LPCs into hepatocytes through the activation of the Notch1 pathway. Genealogy tracking experiment has revealed that Notch–RBPJ signalling is vital in bile duct regeneration and *i*n vitro differentiation of LPCs to cholangiocytes, underscoring the central role of Notch signalling in LPCs differentiation.^90^, ^91^ Furthermore, studies have indicated that the NOTCH–YAP1/TEAD–DNMT1 axis is critical for the transformation of hepatocytes into BECs. The Notch intracellular domain (NICD) independently regulates SOX9 and YAP1. Inhibiting either YAP1 or TEAD can hinder this transdifferentiation. DNMT1, a downstream effector of the YAP1–TEAD complex, directs hepatocyte transformation to BECs by repressing hepatocyte‐specific genes such as HNF‐4α, HNF‐1α and CCAAT/enhancer‐binding proteins α/β. DNMT1 deletion prevents the NOTCH/YAP1‐mediated hepatocyte transdifferentiation to BECs and the resulting cholangiocarcinogenesis. In vivo, time‐lapse imaging has shown that the conversion from hepatocytes to BECs occurs independently of a proliferative intermediate stage^92^ (Figure 5 and Table 2).

**TABLE 2:** Signalling pathways of LPCs differentiation.

Signalling pathway	Role in LPC differentiation	Key interactions and effects	References
Wnt/β‐catenin	Promotes LPC differentiation into hepatocytes	Activation by macrophages phagocytosing hepatocyte debris, leading to hepatocyte differentiation. Inhibition or over‐activation can disrupt biliary tract development.	^34^, ^86^, ^87^
Notch	Regulates proliferation and maintenance of stem/progenitor cells	Involved in bile duct differentiation. Reactivated in BECs during regeneration, aiding in cholangiocyte differentiation from LPCs. EpCAM activates Notch1 to drive differentiation.	^88^, ^89^, ^90^, ^91^, ^92^
HGF/c‐Met	Essential for LPC transformation into hepatocytes	Activates downstream pathways (e.g., Erk1/2, AKT, STAT3) promoting differentiation. C‐Met deficiency impairs this ability.	^53^, ^93^
Hippo/YAP	May regulate LPC differentiation	Ectopic expression of YAP reprograms mature hepatocytes into LPCs, capable of differentiating into hepatocytes and cholangiocytes.	^94^

HGF is crucial for inducing LPCs transformation into hepatocytes. HGF triggers the activation of the c‐Met receptor, leading to the activation of downstream effectors such as Erk1/2, AKT and STAT3, ultimately promoting the differentiation of LPCs. The absence of the c‐Met receptor impairs LPCs proliferation and migration.^53^ HGF/c‐Met‐mediated Akt and STAT3 activation is necessary for differentiation from LPCs to hepatocytes. c‐Met deficiency leads to the loss of LPCs' ability to differentiate into hepatocytes.^93^

The Hippo–YAP pathway is essential for controlling the differentiation of LPCs. YAP ectopically expressed in mature hepatocytes transforms them into LPCs.^94^, ^95^ Recent research has offered a more profound understanding of the intricate systems that underlie this pathway.^94^ For instance, a single‐cell RNA sequencing study revealed significant heterogeneity in YAP expression among cholangiocytes in a 3,5‐diethoxycarbonyl‐1,4‐dihydrocollidine injury model. This heterogeneity indicates that different subsets of cholangiocytes may respond differently to injury and regeneration cues. Additionally, bile acids have been demonstrated to control YAP activation, a crucial factor in preserving biliary reactions in the event of liver damage and recovery.^94^ Further studies have also emphasised the interaction between the Hippo–YAP pathway and various signalling pathways like Wnt and Notch, influencing the behaviour of LPCs and liver regeneration.^96^ The results highlight the significance of the Hippo–YAP pathway in liver function and its promise as a target for treating liver disorders.^96^, ^97^

### 3.4 Cellular interactions in liver injury and regeneration

Liver regeneration primarily focuses on the proliferation of hepatocytes, which requires the coordinated efforts of several NPCs, including LSECs, HSCs and KCs. Interactions among hepatocytes and NPCs form a complex regulatory network, essential for restoring liver mass and function. Understanding these complex intercellular communications is vital for a deeper insight into the regeneration process.

#### 3.4.1 Interaction of KCs with hepatocytes

As previously mentioned, liver regeneration involves interactions between various cell signalling molecules. KCs located between the endothelium of hepatic sinusoids and hepatocytes account for about 80% of the body's total macrophages.^98^ Upon liver injury, increased blood flow activates the synthesis and release of HGF and EGF. Afterwards, KCs are activated by these stimuli to generate and release TNF‐α. TNF‐α functions in an autocrine manner regulated by NF‐κB. NF‐κB also triggers the release of IL‐6. IL‐6 binds to its receptors on the cell surface of hepatocytes, activating the JAK–STAT3 pathway. This enhances the progression of hepatocytes from G1 to S phase by increasing the expression of cyclin D1, thereby promoting cell cycle initiation and replication of hepatocytes.^98^, ^99^ Research has demonstrated that KCs can impede the process of liver regeneration by releasing substances such as TGF‐β.^99^ The communications between KCs and hepatocytes are crucial in liver regeneration.

#### 3.4.2 Interaction of LSECs with hepatocytes

LSECs located in the hepatic sinusoids, are the first to contact hepatic blood flow and are also the earliest cells to be damaged in liver diseases. LSECs, unique endothelial cells with abundant window pore structures and no intact basement membrane constitute approximately 70% of the liver's NPCs.^100^ LSECs secrete diverse cytokines that are crucial for inducing hepatocyte regeneration and maintaining the quiescence of HSCs.^101^ Sources of LSECs in liver regeneration encompass bone marrow‐derived sinusoidal endothelial precursor cells (BM‐SPCs), mature LSECs and resident liver SPCs. Precise synchronisation between LSECs and hepatocytes is essential for liver regeneration. LSECs coordinate the release of cytokines and growth factors to promote hepatocyte proliferation, while hepatocytes also control the proliferation of LSECs. During PHx, there was an increase in hepatic vascular endothelial growth factor expression, leading to the recruitment of HGF‐rich BM‐SPCs through the stromal cell‐derived factor‐1 (SDF‐1)/CXCR7 axis.^102^ Nitric oxide (NO) secreted by LSECs sensitises hepatocytes to HGF, promoting liver regeneration.^103^ During APAP‐induced acute liver injury, LSECs secrete Wnt proteins and enhance HGF expression in the repair phase. This LSECs originated Wnt signal promotes β‐catenin activation in hepatocytes, thus up‐regulating target genes like cyclin D1 and driving hepatocyte proliferation.^104^ In CCl_4_‐induced acute liver injury, LSECs‐mediated activation of c‐Kit pathway facilitates liver repair via Wnt2‐dependent manner.^105^ In studies on NASH, LSECs secrete cytokines and extensively interact with cholangiocytes, HSCs and other NPCs. In LSECs of NASH livers, expression of genes related with lipid metabolism was up‐regulated and those for vascular homeostasis were down‐regulated, resulting in the destruction of capillaries in hepatic sinusoids. LSECs also express DLL4 and TGF‐β1, which interact with corresponding receptors on monocytes, leading to their differentiation into hepatic macrophages.^105^, ^106^

#### 3.4.3 Interaction of HSCs with hepatocytes

HGF is essential for regulating the multiplication of hepatocytes, making it a key growth factor. Hepatocytes receive HGF through both endocrine and paracrine manner.^107^ HSCs that have been activated, located in the Disse space between hepatic sinusoidal endothelial and hepatic epithelial cells, are the main producers of HGF.^108^ During liver injury and other pathological conditions, HGF binds directly to c‐MET, a hepatocyte surface receptor, initiating hepatocyte proliferation. HGF's binding to c‐MET activates downstream pathways such as the MAPK cascade, PI3K–Akt–STAT3 axis and NF‐κB pathway.^109^ During early liver regeneration stages, HSCs produce norepinephrine. This reduces the inhibitory impact of TGF‐β on mitosis and boosts HGF and EGF secretion.^110^ During the terminal phase of regeneration, HSCs separate excess growth factors by reorganising the ECM, causing hepatocytes to stop dividing.^111^ Concurrently, the activation and differentiation of LPCs, as previously mentioned, occur amidst multiple cellular interactions. Damaged hepatocytes directly activate HSCs. Meanwhile, macrophages, monocytes, T‐lymphocytes and hepatic sinusoidal endothelial cells modulate HSCs activity by secreting factors like IL‐6, TNF‐α, HGF and TGF‐α/β.^112^, ^113^ These intercellular interactions and signals uncover complex regenerative mechanisms in the liver after injury (Table 3).

**TABLE 3:** Cellular interactions in liver regeneration.

Cell or cellular component	Involved cytokines/pathways	Relationship to other cells	References
KCs	TNF‐α, IL‐6, TGF‐β, JAK–STAT3 pathway	Promotes hepatocyte proliferation via cell cycle proteins; also inhibits hepatocyte regeneration	^98^, ^99^
LSECs	HGF, NO, DLL4, TGF‐β1, Wnt/β‐catenin pathway, c‐kit pathway	Enhances hepatocyte response to HGF; promotes hepatocyte replication via cell cycle proteins; activates stem cells; promotes monocyte differentiation	^100^, ^101^, ^102^, ^103^, ^104^, ^105^, ^106^
HSCs	HGF, EGF, c‐MET, MAPK pathway, PI3K–Akt–STAT3 pathway, NF‐kb pathway	Generation of HGF to promote hepatocyte proliferation; remodelling of ECM to terminate regeneration	^107^, ^108^, ^109^, ^110^, ^111^, ^112^, ^113^
ECM	Integrin signalling, MAPK pathway, PI3K‐Akt‐STAT3 pathway, Wnt/β‐catenin pathway	Affects hepatocyte proliferation and migration; regulates immune cell behaviour and macrophage phenotype via cytokines and chemotactic factors	^114^, ^115^, ^116^, ^117^, ^118^, ^119^

#### 3.4.4 Interaction of ECM with hepatocytes

Upon liver injury, the composition of the ECM undergoes significant changes, and these changes have a profound effect on hepatocyte behaviour and liver regenerative capacity. For example, increased deposition of collagen and elastin enhances ECM stiffness, which in turn affects hepatocyte proliferation and migration.^114^ The ECM is not only a passive structural framework but also regulates intracellular signalling pathways by binding to cell‐surface integrins.^115^ It has been shown that increased ECM stiffness can activate multiple intracellular signals, including MAPK, PI3K/Akt and Wnt/β‐catenin pathways.^116^, ^117^ Changes in the ECM during liver injury affect not only hepatocytes but also the behaviour of immune cells such as KCs. These cells can secrete cytokines and chemotactic factors that further regulate the composition and function of the ECM, forming a complex network of interactions. For example, TGF‐β produced by macrophages can stimulate HSCs to produce more collagen and exacerbate hepatic fibrosis, and altering the structure of the ECM can also influence macrophage phenotype and function.^27^, ^118^, ^119^

## 4 INTERVENTIONS AND THERAPEUTIC PERSPECTIVES IN LIVER REGENERATION

Over the past few decades, there has been a rise in groundbreaking technologies focused on liver regeneration, presenting new treatment possibilities for a range of liver conditions. These technologies range from bioartificial systems that mimic liver functions, to advanced methods like normothermic machine perfusion (NMP) that preserve liver integrity before transplantation. Alongside, nanomaterials are being explored for their therapeutic potential, while stem cell technologies pave the way for cell replacement therapies. Additionally, organoids provide a novel approach for in vivo liver regeneration, showcasing how these advancements collectively push the boundaries of medical science towards effective liver treatment and recovery.

### 4.1 Normothermic machine perfusion

NMP maintains liver activity prior to transplantation by supplying oxygen and nutrient‐rich blood through a circulatory system that mimics that of the human body, keeping the liver functioning at body temperature.^120^ NMP helps to reduce the duration of oxygen deprivation, optimise metabolic status and reduce the risk of liver damage after transplantation.^120^, ^121^ Studies have shown that NMP significantly improves the success rate of transplantation of borderline quality livers, especially from cardiac arrest donors.^122^, ^123^ NMP can be utilised for drug testing and metabolic research to evaluate the possibility of liver function and injury recovery, as well as to expand the pool of livers for transplantation.^123^

### 4.2 Nanomaterials

Nanomaterials, with their unique properties such as small size, surface and quantum effects, are considered potent tools for treating liver diseases. They were used for drug delivery, imaging and as scaffolds for cell growth.^124^ Nanoparticles made of cerium oxide, which are effective at removing reactive oxygen species, greatly speed up the process of liver regeneration in rats that have experienced liver injury from post PHx and APAP overdose. These nanoparticles induce hepatocyte proliferation, reduce stress markers, advance cell cycle progression and activate NF‐κB transcription factors.^125^ Tetrahedral framework nucleic acids (TFNAs), known for their antioxidant and anti‐inflammatory properties, activate various proliferative and prosurvival pathways. TFNAs stimulate hepatocyte proliferation and liver repair in mouse models of 70% PHx, APAP overdose and CCl_4_ injury by triggering the Notch, Wnt and P53 pathways.^126^ Additionally, a study highlighted the potential of using magnetic nanoparticles to attract stem cells to migrate to damaged liver areas.^127^ These findings indicate that nanomaterials and nanostructures hold substantial potential for enhancing liver regeneration and offering new therapeutic options for liver disease.

### 4.3 Stem cell‐related technologies

Stem cell‐related technologies, particularly pluripotent and adult stem cells, present a promising approach for cell replacement and liver regeneration. Stem cells, capable of differentiating into hepatocytes, also play vital roles in liver preservation through anti‐inflammatory, immunosuppressive, angiogenic and anti‐apoptotic mechanisms. Additionally, research indicates that paracrine factors from stem cells promote angiogenesis, decrease inflammation and inhibit hepatocyte apoptosis.^127^ Various stem cells, especially mesenchymal stem cells (MSCs), are used in liver therapy. MSCs are derived from adult or perinatal tissues like bone marrow, placenta, umbilical cord, liver and adipose tissue. In a CCl_4_ liver injury model, stem cells from umbilical cord blood successfully differentiate into hepatocyte‐like cells, thus ameliorate liver damage levels and fibrosis degree and also restoring liver functions evidenced by decreased aminotransferase and increased albumin levels.^128^ Transplanting induced MSCs derived from embryonic stem cells in rats post PHx reduce bilirubin levels and promote liver regeneration in vivo.

Recent studies have highlighted the role of MSC‐derived or bone marrow MSC‐derived extracellular vesicles (EVs) as novel therapeutic agents.^129^, ^130^ These EVs can package and deliver bioactive molecules such as RNA, DNA, proteins and lipids that modulate inflammation, reduce fibrosis and promote tissue regeneration.^131^, ^132^ This cell‐free treatment may offer a safer alternative to direct stem cell transplantation, reducing the risk of cell integration and proliferation.^132^

### 4.4 Organoids

Organoids, a recently developed biological model, have become instrumental in liver regeneration research. They are three‐dimensional, organ‐like structures generated from stem or organ‐specific cells cultured in vitro.^133^ Organoids, when grafted in vivo, can connect to host blood vessels through vascularisation, maturing more effectively than in vitro.^134^ Researchers created liver‐like organs (LOs) with hiPSC‐derived endoderm, endothelial cells and mesenchymal stromal cells from a single donor and transplanted the LOs into mice with acute liver failure. The transplant mice showed improved liver function and increased survival rates in a short time.^135^ Primary cells isolated from rat livers can expand chronically into organoids or LPCs under the induction of Wnt agonists, TGF‐β inhibitors or the injury‐induced inflammatory cytokine TNF‐α. Under these conditions, rat hepatocyte derived organoids were able to expand in vitro for approximately 2−3 months and express bile duct markers as well as LPCs markers. Notably, these expanded rat hepatocyte‐like organs recapitulate the proliferation pattern of hepatocytes after PHx in terms of gene expression pattern.^136^, ^137^ A research study documented the creation of functional hepatobiliary organoids from hepatocytes.^138^ This group of organoids consists of a system of bile ducts encircled by fully developed hepatocytes and effectively preserves hepatic traits and functionality both in laboratory settings and following transplantation into living organisms.^138^ Inhibiting Tead4 or increasing Ddit3 expression can reverse hepatocyte fate decisions during in vitro and in vivo transplantation, uncovering the key transcription factors that control hepatobiliary cell fate determination in the liver.^138^ In addition, it has been reported that adult hepatocytes can be induced into LPCs in vitro and expanded in long‐term culture under certain culture conditions. However, when these expanded LPCs were further cultured into organoids, they redifferentiated into mature hepatocytes and showed significant improvement in liver function as well as a significant reduction in hepatic progenitor cell characteristics.^84^, ^139^

Currently, it is possible to construct proliferating human hepatocytes (ProliHHs) by dedifferentiating primary human hepatocytes to a dual phenotype state without genetic manipulation.^140^, ^141^ ProliHHs have hepatocyte and progenitor cell characteristics, that can expand at least 10,000‐fold, and, in a 3D organoid system able to redifferentiate to a mature state close to primary human hepatocytes.^140^ In ProliHH‐like organs, hepatic gene expression and corresponding liver function are significantly improved.^141^ Intraperitoneal transplantation of encapsulated ProliHHs liver organoids (eLO) was performed on animals with liver failure.^142^, ^143^ In a mouse model of liver failure after 80% hepatectomy, eLO significantly improved survival and provided normal liver function, resulting in amelioration of hyperammonaemia and hypoglycaemia.^144^ Furthermore, eLO therapy safeguarded the gut barrier, decreased endotoxin levels and suppressed inflammation, ultimately facilitating the regeneration of the liver. The effectiveness of the treatment was also validated in a mouse model with excessive APAP‐induced liver failure.^144^ And eLO caused no adverse effects in mice and was consistently non‐tumourigenic.^144^ Treatment of two mouse models of liver failure demonstrated the efficacy of eLO intraperitoneal transplantation and confirmed the safety of eLO, providing a solid foundation for ongoing organoid clinical studies. It is promising that in the future, doctors may use patient‐specific cells to culture organoids, enabling personalised treatment regimens. This approach mitigates sample availability and ethical concerns associated with allogeneic transplantation and lessens the need for immunosuppressive drugs in patients.

Cholangiocyte organoids are miniature biostructures generated in vitro using stem cell technology that mimic the physiological function and structure of real bile ducts.^145^ These organoids can be applied for bile duct repair after liver transplantation. Cholangiocyte organoids can be established by inducing differentiation of human pluripotent stem cells, such as induced pluripotent stem cells (iPSCs) or adult stem cells, under specific culture conditions.^146^, ^147^ Studies demonstrated the possibility of using human cholangiocyte organoids to repair damaged bile ducts.^145^, ^147^ The researchers proved that human iPSCs‐derived cholangiocyte organoids not only possess normal bile duct function in vitro, but also promote repair and regeneration of the damaged bile ducts after transplanted into the damaged bile duct region.^146^, ^147^

### 4.5 Bioartificial liver

The bioartificial liver (BAL) system is a device that uses living cells to simulate liver functions, providing temporary life support to patients with acute or chronic liver failure.^148^ Recent research developments have shown that these systems can not only filter toxins from the blood but also secrete liver‐specific proteins such as albumin and transferrin, potentially facilitating liver regeneration.^149^, ^150^

Presently, BAL systems predominantly employ two types of hepatic cells: primary hepatocytes and stem cell‐derived hepatocytes.^151^ Primary hepatocytes are typically sourced from human or animal livers, but the difficult availability and variable quality limit their application. Consequently, increasing researchers are utilising human pluripotent stem cells derived hepatocytes, which express hepatic marker genes and possess liver cell functions, such as protein synthesis, urea production and glycogen storage.^152^

Animal model studies have demonstrated that the BAL system significantly improved survival rates and liver functions in acute liver failure.^153^ More inspiringly, the BAL system has shown promising results in clinical trials, that treating acute liver failure patients with the BAL system safely improved their immunoglobulin levels and significantly enhanced short‐term survival rates.^154^ With technological advancements and the accumulation of clinical data, the BAL system is poised to become an effective supplement or alternative to liver transplantation, particularly where there is a shortage of liver donors.

The advancements in liver regeneration technologies signify a monumental shift in therapy for liver diseases. From the precision of nanomaterials in drug delivery and liver repair to the sophisticated developments in organoid and BAL systems, these technologies collectively enhance our capability to treat and manage liver repair more effectively. As research progresses, these innovations are promise to improve liver therapies and broaden the scope of recoverable liver conditions, ultimately improving patients’ outcomes and life quality.

## 5 DISCUSSION

In the current study, we have thoroughly explored various aspects of liver regeneration, particularly the liver's self‐repair capabilities following acute and chronic liver injury. While several signalling pathways, including the Wnt/β‐catenin, Notch and Hippo/YAP pathways, have been linked to liver regeneration, the precise functions and interplay of these pathways in various liver injury types are still not well known. The Wnt/β‐catenin pathway, for example, is essential for liver formation and regeneration, but more research is needed to understand how it regulates specific liver diseases and how it affects LPC behaviour. Furthermore, although the Notch pathway is important in controlling the plasticity between cholangiocytes and hepatocytes, little is known about how it contributes to the development of liver fibrosis and whether it has any potential therapeutic uses.

Further research is necessary to fully understand the liver's capacity for regeneration in individuals suffering from chronic liver disorders such cirrhosis and liver fibrosis. In these cases, the liver's ability to regenerate is severely restricted, and exploring how to activate and enhance this capability without causing liver cancer is a crucial direction for future research. Meanwhile, although the study of LPCs has provided a new perspective on liver regeneration, their specific roles and potential in different types of liver injuries still need further exploration. For example, the mechanisms behind the activation and differentiation of LPCs following chronic liver injury and how these mechanisms are influenced by the surrounding microenvironment remain current research hotspots.

Approaches for enhancing liver regrowth with stem cells and organoid technology have displayed potential, yet substantial obstacles need to be addressed for successful implementation in a clinical setting. For instance, ensuring that liver cells regenerated through these methods have adequate functionality and long‐term stability, as well as avoiding potential immune rejection or malignant transformation, are key issues that future research must address.

Further studies should concentrate on uncovering the molecular processes involved in liver regeneration and how this information can assist in treating liver disorders. The increasing incidence of CLDs and other liver conditions urgently demands new treatment strategies. We anticipate progress in treating liver diseases with ongoing technological advancements and research developments.

## AUTHOR CONTRIBUTIONS

Xiaofang Zhao and Hongyang Wang planned the project. Jing Xu, Wenjuan Wei and Hao Song collected relevant information. Yao Chen, Jing Fu and Senyan Wang determined the structure of the article. All authors analysed the data. Qi Liu wrote the paper. All authors discussed the results and commented on the manuscript.

## CONFLICT OF INTEREST STATEMENT

The authors declare no conflict of financial interests.

## ETHICS STATEMENT

Not applicable.

## Data Availability

All relevant data are available from the authors upon request.

## References

[1] Trefts E , Gannon M , Wasserman DH . The liver. Curr Biol. 2017;27:R1147‐r1151. doi:10.1016/j.cub.2017.09.019 29112863 PMC5897118

[2] Campana L , Esser H , Huch M , Forbes S . Liver regeneration and inflammation: from fundamental science to clinical applications. Nat Rev Mol Cell Biol. 2021;22:608‐624. doi:10.1038/s41580-021-00373-7 34079104

[3] Michalopoulos GK , Bhushan B . Liver regeneration: biological and pathological mechanisms and implications. Nat Rev Gastroenterol Hepatol. 2021;18:40‐55. doi:10.1038/s41575-020-0342-4 32764740

[4] Hu Y , Wang R , An Ni , et al. Unveiling the power of microenvironment in liver regeneration: an in‐depth overview. Front Genet. 2023;14:1332190. doi:10.3389/fgene.2023.1332190 38152656 PMC10751322

[5] Paris J , Henderson NC . Liver zonation, revisited. Hepatology. 2022;76:1219‐1230. doi:10.1002/hep.32408 35175659 PMC9790419

[6] Michalopoulos GK . Liver regeneration. J Cell Physiol. 2007;213:286‐300. doi:10.1002/jcp.21172 17559071 PMC2701258

[7] Li W , Li Lu , Hui L . Cell plasticity in liver regeneration. Trends Cell Biol. 2020;30:329‐338. doi:10.1016/j.tcb.2020.01.007 32200807

[8] Rizvi F , Lee Yu‐Ri , Diaz‐Aragon R , et al. VEGFA mRNA‐LNP promotes biliary epithelial cell‐to‐hepatocyte conversion in acute and chronic liver diseases and reverses steatosis and fibrosis. Cell Stem Cell. 2023;30:1640‐1657.e1648. doi:10.1016/j.stem.2023.10.008 38029740 PMC10843608

[9] Wang B , Shen H , Wei Y , et al. Balance of Gata3 and Ramp2 in hepatocytes regulates hepatic vascular reconstitution in postoperative liver regeneration. J Hepatol. 2024;80:309‐321. doi:10.1016/j.jhep.2023.10.016 37918568

[10] Nicolas CT , Hickey RD , Chen HS , et al. Concise review: liver regenerative medicine: from hepatocyte transplantation to bioartificial livers and bioengineered grafts. Stem Cells. 2017;35:42‐50. doi:10.1002/stem.2500 27641427 PMC5529050

[11] Gao Ce , Peng J . All routes lead to Rome: multifaceted origin of hepatocytes during liver regeneration. Cell Regen. 2021;10:2. doi:10.1186/s13619-020-00063-3 33403526 PMC7785766

[12] Yagi S , Hirata M , Miyachi Y , Uemoto S . Liver regeneration after hepatectomy and partial liver transplantation. Int J Mol Sci. 2020;21. doi:10.3390/ijms21218414 PMC7665117 33182515

[13] Wu Yi , Li N , Shu X , et al. Biomechanics in liver regeneration after partial hepatectomy. Front Bioeng Biotechnol. 2023;11:1165651. doi:10.3389/fbioe.2023.1165651 37214300 PMC10196191

[14] Taub R . Liver regeneration: from myth to mechanism. Nat Rev Mol Cell Biol. 2004;5:836‐847. doi:10.1038/nrm1489 15459664

[15] Higgins GM . Experimental pathology of the liver. 12. Effect of feeding desiccated thyroid gland on restoration of the liver. Arch Pathol. 1933;16:226‐231.

[16] Van Haele M , Snoeck J , Roskams T . Human liver regeneration: an etiology dependent process. Int J Mol Sci. 2019;20. doi:10.3390/ijms20092332 PMC6539121 31083462

[17] Gilgenkrantz H , Collin De l'Hortet A . Understanding liver regeneration: from mechanisms to regenerative medicine. Am J Pathol. 2018;188:1316‐1327. doi:10.1016/j.ajpath.2018.03.008 29673755

[18] Bonnardel J , T'jonck W , Gaublomme D , et al. Stellate cells, hepatocytes, and endothelial cells imprint the kupffer cell identity on monocytes colonizing the liver macrophage niche. Immunity. 2019;51:638‐654. doi:10.1016/j.immuni.2019.08.017 e639 31561945 PMC6876284

[19] Kawasaki S , Makuuchi M , Ishizone S , Matsunami H , Terada M , Kawarazaki H . Liver regeneration in recipients and donors after transplantation. Lancet. 1992;339:580‐581. doi:10.1016/0140-6736(92)90867-3 1347095

[20] Alison MR . Regulation of hepatic growth. Physiol Rev. 1986;66:499‐541. doi:10.1152/physrev.1986.66.3.499 2426724

[21] Kwon YJ , Lee KG , Choi D . Clinical implications of advances in liver regeneration. Clin Mol Hepatol. 2015;21:7‐13. doi:10.3350/cmh.2015.21.1.7 25834796 PMC4379199

[22] Jaeschke H , Akakpo JY , Umbaugh DS , Ramachandran A . Novel therapeutic approaches against acetaminophen‐induced liver injury and acute liver failure. Toxicol Sci. 2020;174:159‐167. doi:10.1093/toxsci/kfaa002 31926003 PMC7098369

[23] Chao X , Wang H , Jaeschke H , Ding W‐X . Role and mechanisms of autophagy in acetaminophen‐induced liver injury. Liver Int. 2018;38:1363‐1374. doi:10.1111/liv.13866 29682868 PMC6105454

[24] Lukacs‐Kornek V , Lammert F . The progenitor cell dilemma: cellular and functional heterogeneity in assistance or escalation of liver injury. J Hepatol. 2017;66:619‐630. doi:10.1016/j.jhep.2016.10.033 27826058

[25] Faccioli LAP , Dias ML , Paranhos BA , Dos Santos Goldenberg RC . Liver cirrhosis: an overview of experimental models in rodents. Life Sci. 2022;301:120615. doi:10.1016/j.lfs.2022.120615 35526595

[26] Iredale JP . Models of liver fibrosis: exploring the dynamic nature of inflammation and repair in a solid organ. J Clin Investig. 2007;117:539‐548. 17332881 10.1172/JCI30542 PMC1804370

[27] Tacke F , Zimmermann HW . Macrophage heterogeneity in liver injury and fibrosis. J Hepatol. 2014;60:1090‐1096. doi:10.1016/j.jhep.2013.12.025 24412603

[28] Ko S , Russell JO , Tian J , et al. Hdac1 Regulates differentiation of bipotent liver progenitor cells during regeneration via Sox9b and Cdk8. Gastroenterology. 2019;156:187‐202. doi:10.1053/j.gastro.2018.09.039 e114 30267710 PMC6309465

[29] Russell JO , Lu W‐Yu , Okabe H , et al. Hepatocyte‐specific β‐catenin deletion during severe liver injury provokes cholangiocytes to differentiate into hepatocytes. Hepatology. 2019;69:742‐759. doi:10.1002/hep.30270 30215850 PMC6351199

[30] Gu CY , Lee TKW . Preclinical mouse models of hepatocellular carcinoma: an overview and update. Exp Cell Res. 2022;412:113042. doi:10.1016/j.yexcr.2022.113042 35101391

[31] Tao Y , Wang M , Chen E , Tang H . Liver regeneration: analysis of the main relevant signaling molecules. Mediators Inflamm. 2017;2017:4256352. doi:10.1155/2017/4256352 28947857 PMC5602614

[32] Tsagianni A , Mars WM , Bhushan B , et al. Combined systemic disruption of MET and epidermal growth factor receptor signaling causes liver failure in normal mice. Am J Pathol. 2018;188:2223‐2235. doi:10.1016/j.ajpath.2018.06.009 30031724 PMC6168971

[33] Kaminsky‐Kolesnikov Y , Rauchbach E , Abu‐Halaka D , et al. Cholesterol induces Nrf‐2‐ and HIF‐1α‐dependent hepatocyte proliferation and liver regeneration to ameliorate bile acid toxicity in mouse models of NASH and fibrosis. Oxid Med Cell Longev. 2020;2020:5393761. doi:10.1155/2020/5393761 32566088 PMC7271232

[34] Apte U , Thompson MD , Cui S , Liu B , Cieply B , Monga SPS . Wnt/beta‐catenin signaling mediates oval cell response in rodents. Hepatology. 2008;47:288‐295. doi:10.1002/hep.21973 17929301

[35] Jin X , Zimmers TA , Jiang Y , Milgrom DP , Zhang Z , Koniaris LG . Meloxicam increases epidermal growth factor receptor expression improving survival after hepatic resection in diet‐induced obese mice. Surgery. 2018;163:1264‐1271. doi:10.1016/j.surg.2017.11.029 29361369 PMC5985213

[36] Zwirner S , Abu Rmilah AA , Klotz S , et al. First‐in‐class MKK4 inhibitors enhance liver regeneration and prevent liver failure. Cell. 2024;187:1666‐1684. doi:10.1016/j.cell.2024.02.023 e1626 38490194 PMC11011246

[37] Nejak‐Bowen KN , Monga SPS . Beta‐catenin signaling, liver regeneration and hepatocellular cancer: sorting the good from the bad. Semin Cancer Biol. 2011;21:44‐58. doi:10.1016/j.semcancer.2010.12.010 21182948 PMC3050081

[38] Yang W , Yan He‐X , Chen L , et al. Wnt/beta‐catenin signaling contributes to activation of normal and tumorigenic liver progenitor cells. Cancer Res. 2008;68:4287‐4295. doi:10.1158/0008-5472.Can-07-6691 18519688

[39] Li N , Kong M , Zeng S , et al. Brahma related gene 1 (Brg1) contributes to liver regeneration by epigenetically activating the Wnt/β‐catenin pathway in mice. Faseb J. 2019;33:327‐338. doi:10.1096/fj.201800197R 30001167

[40] Zhu Y , Qiu Z , Zhang Y , Li B , Jiang X . Partial hepatectomy‑induced upregulation of SNHG12 promotes hepatocyte proliferation and liver regeneration. Mol Med Rep. 2020;21:1089‐1096. doi:10.3892/mmr.2019.10904 31894329 PMC7003022

[41] Yang J , Mowry LE , Nejak‐Bowen KN , et al. β‐catenin signaling in murine liver zonation and regeneration: a Wnt‐Wnt situation!. Hepatology. 2014;60:964‐976. doi:10.1002/hep.27082 24700412 PMC4139486

[42] Russell JO , Monga SP . Wnt/β‐catenin signaling in liver development, homeostasis, and pathobiology. Annu Rev Pathol. 2018;13:351‐378. doi:10.1146/annurev-pathol-020117-044010 29125798 PMC5927358

[43] Aldeguer X , Debonera F , Shaked A , et al. Interleukin‐6 from intrahepatic cells of bone marrow origin is required for normal murine liver regeneration. Hepatology. 2002;35:40‐48. doi:10.1053/jhep.2002.30081 11786958

[44] Schmidt‐Arras D , Rose‐John S . IL‐6 pathway in the liver: from physiopathology to therapy. J Hepatol. 2016;64:1403‐1415. doi:10.1016/j.jhep.2016.02.004 26867490

[45] Sun J‐Y , Du L‐J , Shi X‐R , et al. An IL‐6/STAT3/MR/FGF21 axis mediates heart‐liver cross‐talk after myocardial infarction. Sci Adv. 2023;9:eade4110. doi:10.1126/sciadv.ade4110 37018396 PMC10075967

[46] Johnson DE , O'keefe RA , Grandis JR . Targeting the IL‐6/JAK/STAT3 signalling axis in cancer. Nat Rev Clin Oncol. 2018;15:234‐248. doi:10.1038/nrclinonc.2018.8 29405201 PMC5858971

[47] Park J , Zhao Y , Zhang F , et al. IL‐6/STAT3 axis dictates the PNPLA3‐mediated susceptibility to non‐alcoholic fatty liver disease. J Hepatol. 2023;78:45‐56. doi:10.1016/j.jhep.2022.08.022 36049612 PMC9772150

[48] Hunter CA , Jones SA . IL‐6 as a keystone cytokine in health and disease. Nat Immunol. 2015;16:448‐457. doi:10.1038/ni.3153 25898198

[49] Roxburgh CSD , Mcmillan DC . Therapeutics targeting innate immune/inflammatory responses through the interleukin‐6/JAK/STAT signal transduction pathway in patients with cancer. Transl Res. 2016;167:61‐66. doi:10.1016/j.trsl.2015.08.013 26432924

[50] Van Snick J . Interleukin‐6: an overview. Annu Rev Immunol. 1990;8:253‐278. doi:10.1146/annurev.iy.08.040190.001345 2188664

[51] Wen Y , Emontzpohl C , Xu L , et al. Interleukin‐33 facilitates liver regeneration through serotonin‐involved gut‐liver axis. Hepatology. 2023;77:1580‐1592. doi:10.1002/hep.32744 36129070 PMC10758291

[52] Huang M , Jiao J , Cai H , et al. C‐C motif chemokine ligand 5 confines liver regeneration by down‐regulating reparative macrophage‐derived hepatocyte growth factor in a forkhead box O 3a‐dependent manner. Hepatology. 2022;76:1706‐1722. doi:10.1002/hep.32458 35288960 PMC9790589

[53] Ishikawa T , Factor VM , Marquardt JU , et al. Hepatocyte growth factor/c‐met signaling is required for stem‐cell‐mediated liver regeneration in mice. Hepatology. 2012;55:1215‐1226. doi:10.1002/hep.24796 22095660 PMC3299882

[54] Péan N , Doignon I , Garcin I , et al. The receptor TGR5 protects the liver from bile acid overload during liver regeneration in mice. Hepatology. 2013;58:1451‐1460. doi:10.1002/hep.26463 23686672

[55] Doignon I , Julien B , Serrière‐Lanneau V , et al. Immediate neuroendocrine signaling after partial hepatectomy through acute portal hyperpressure and cholestasis. J Hepatol. 2011;54:481‐488. doi:10.1016/j.jhep.2010.07.012 21163545

[56] Wang Y‐D , Chen W‐D , Moore DD , Huang W . FXR: a metabolic regulator and cell protector. Cell Res. 2008;18:1087‐1095. doi:10.1038/cr.2008.289 18825165

[57] Huang W , Ma Ke , Zhang J , et al. Nuclear receptor‐dependent bile acid signaling is required for normal liver regeneration. Science. 2006;312:233‐236. doi:10.1126/science.1121435 16614213

[58] Frampton G , Reddy P , Jefferson B , Ali M , Khan D , Mcmillin M . Inhibition of thrombospondin‐1 reduces glutathione activity and worsens acute liver injury during acetaminophen hepatotoxicity in mice. Toxicol Appl Pharmacol. 2020;409:115323. doi:10.1016/j.taap.2020.115323 33176120 PMC8364670

[59] Sivilotti MLA , Yarema MC , Juurlink DN . Treating acetaminophen overdose. Cmaj. 2022;194:E554. doi:10.1503/cmaj.210703 35440504 PMC9035297

[60] Chidiac AS , Buckley NA , Noghrehchi F , Cairns R . Paracetamol (acetaminophen) overdose and hepatotoxicity: mechanism, treatment, prevention measures, and estimates of burden of disease. Expert Opin Drug Metab Toxicol. 2023;19:297‐317. doi:10.1080/17425255.2023.2223959 37436926

[61] Chiew AL , Gluud C , Brok J , Buckley NA . Interventions for paracetamol (acetaminophen) overdose. Cochrane Database Syst Rev. 2018;2:Cd003328. doi:10.1002/14651858.CD003328.pub3 29473717 PMC6491303

[62] Viswanathan P , Sharma Y , Gupta P , Gupta S . Replicative stress and alterations in cell cycle checkpoint controls following acetaminophen hepatotoxicity restrict liver regeneration. Cell Prolif. 2018;51:e12445. doi:10.1111/cpr.12445 29504225 PMC6500460

[63] Borude P , Bhushan B , Gunewardena S , Akakpo J , Jaeschke H , Apte U . Pleiotropic role of p53 in injury and liver regeneration after acetaminophen overdose. Am J Pathol. 2018;188:1406‐1418. doi:10.1016/j.ajpath.2018.03.006 29654721 PMC5971235

[64] Xu P , Xi Y , Wang P , et al. Inhibition of p53 sulfoconjugation prevents oxidative hepatotoxicity and acute liver failure. Gastroenterology. 2022;162:1226‐1241. doi:10.1053/j.gastro.2021.12.260 34954226 PMC8934304

[65] Dong S , Ji J , Hu L , Wang H . Dihydromyricetin alleviates acetaminophen‐induced liver injury via the regulation of transformation, lipid homeostasis, cell death and regeneration. Life Sci. 2019;227:20‐29. doi:10.1016/j.lfs.2019.04.019 30974116

[66] Wen Y , Wang C , Gu J , et al. Metabolic modulation of acetaminophen‐induced hepatotoxicity by osteopontin. Cell Mol Immunol. 2019;16:483‐494. doi:10.1038/s41423-018-0033-z 29735980 PMC6474212

[67] Kotulkar M , Paine‐Cabrera D , Abernathy S , et al. Role of HNF4alpha‐cMyc interaction in liver regeneration and recovery after acetaminophen‐induced acute liver injury. Hepatology. 2023;78:1106‐1117. doi:10.1097/hep.0000000000000367 37021787 PMC10523339

[68] Matchett KP , Wilson‐Kanamori JR , Portman JR , et al. Multimodal decoding of human liver regeneration. Nature. 2024;630:158‐165. doi:10.1038/s41586-024-07376-2 38693268 PMC11153152

[69] Lucey MR , Mathurin P , Morgan TR . Alcoholic hepatitis. N Engl J Med. 2009;360:2758‐2769. doi:10.1056/NEJMra0805786 19553649

[70] Sehrawat TS , Liu M , Shah VH . The knowns and unknowns of treatment for alcoholic hepatitis. Lancet Gastroenterol Hepatol. 2020;5:494‐506. doi:10.1016/s2468-1253(19)30326-7 32277902 PMC7238289

[71] Kwo PY . Alcoholic hepatitis and its many facets. Clin Liver Dis. 2021;25:xiii‐xiv. doi:10.1016/j.cld.2021.04.003 34229847

[72] Hosseini N , Shor J , Szabo G . Alcoholic hepatitis: a review. Alcohol. 2019;54:408‐416. doi:10.1093/alcalc/agz036 PMC6671387 31219169

[73] Buzzetti E , Pinzani M , Tsochatzis EA . The multiple‐hit pathogenesis of non‐alcoholic fatty liver disease (NAFLD). Metabolism. 2016;65:1038‐1048. doi:10.1016/j.metabol.2015.12.012 26823198

[74] Samuel VT , Shulman GI . Nonalcoholic fatty liver disease as a nexus of metabolic and hepatic diseases. Cell Metab. 2018;27:22‐41. doi:10.1016/j.cmet.2017.08.002 28867301 PMC5762395

[75] Seeger C , Mason WS . Hepatitis B virus biology. Microbiol Mol Biol Rev. 2000;64:51‐68. doi:10.1128/mmbr.64.1.51-68.2000 10704474 PMC98986

[76] Rehermann B . Hepatitis C virus versus innate and adaptive immune responses: a tale of coevolution and coexistence. J Clin Invest. 2009;119:1745‐1754. doi:10.1172/jci39133 19587449 PMC2701885

[77] Mahmood A , Seetharaman R , Kshatriya P , Patel D , Srivastava AS . Stem cell transplant for advanced stage liver disorders: current scenario and future prospects. Curr Med Chem. 2020;27:6276‐6293. doi:10.2174/0929867326666191004161802 31584360

[78] Gribben C , Galanakis V , Calderwood A , et al. Acquisition of epithelial plasticity in human chronic liver disease. Nature. 2024;630:166‐173. doi:10.1038/s41586-024-07465-2 38778114 PMC11153150

[79] Raven A , Lu W‐Yu , Man TY , et al. Cholangiocytes act as facultative liver stem cells during impaired hepatocyte regeneration. Nature. 2017;547:350‐354. doi:10.1038/nature23015 28700576 PMC5522613

[80] Pu W , Zhu H , Zhang M , et al. Bipotent transitional liver progenitor cells contribute to liver regeneration. Nat Genet. 2023;55:651‐664. doi:10.1038/s41588-023-01335-9 36914834 PMC10101857

[81] Wu H , Zhou Xu , Fu G‐Bo , et al. Reversible transition between hepatocytes and liver progenitors for in vitro hepatocyte expansion. Cell Res. 2017;27:709‐712. doi:10.1038/cr.2017.47 28374751 PMC5520858

[82] Tarlow BD , Pelz C , Naugler WE , et al. Bipotential adult liver progenitors are derived from chronically injured mature hepatocytes. Cell Stem Cell. 2014;15:605‐618. doi:10.1016/j.stem.2014.09.008 25312494 PMC4254170

[83] Huang W‐J , Zhou Xu , Fu G‐Bo , et al. The combined induction of liver progenitor cells and the suppression of stellate cells by small molecules reverts chronic hepatic dysfunction. Theranostics. 2021;11:5539‐5552. doi:10.7150/thno.54457 33859762 PMC8039967

[84] Fu G‐Bo , Huang W‐J , Zeng M , et al. Expansion and differentiation of human hepatocyte‐derived liver progenitor‐like cells and their use for the study of hepatotropic pathogens. Cell Res. 2019;29:8‐22. doi:10.1038/s41422-018-0103-x 30361550 PMC6318298

[85] Liu W‐M , Zhou Xu , Chen C‐Y , et al. Establishment of functional liver spheroids from human hepatocyte‐derived liver progenitor‐like cells for cell therapy. Front Bioeng Biotechnol. 2021;9:738081. doi:10.3389/fbioe.2021.738081 34858956 PMC8630579

[86] Segal JM , Kent D , Wesche DJ , et al. Single cell analysis of human foetal liver captures the transcriptional profile of hepatobiliary hybrid progenitors. Nat Commun. 2019;10:3350. doi:10.1038/s41467-019-11266-x 31350390 PMC6659636

[87] So J , Khaliq M , Evason K , et al. Wnt/β‐catenin signaling controls intrahepatic biliary network formation in zebrafish by regulating notch activity. Hepatology. 2018;67:2352‐2366. doi:10.1002/hep.29752 29266316 PMC5991997

[88] Martinez Lyons A , Boulter L . NOTCH signalling—a core regulator of bile duct disease? Dis Model Mech. 2023;16. doi:10.1242/dmm.050231 PMC10461466 37605966

[89] Boulter L , Govaere O , Bird TG , et al. Macrophage‐derived Wnt opposes Notch signaling to specify hepatic progenitor cell fate in chronic liver disease. Nat Med. 2012;18:572‐579. doi:10.1038/nm.2667 22388089 PMC3364717

[90] Lu J , Zhou Y , Hu T , et al. Notch signaling coordinates progenitor cell‐mediated biliary regeneration following partial hepatectomy. Sci Rep. 2016;6:22754. doi:10.1038/srep22754 26951801 PMC4782135

[91] Tang D , Chen Yi , Fu G‐Bo , et al. EpCAM inhibits differentiation of human liver progenitor cells into hepatocytes in vitro by activating Notch1 signaling. Biochem Biophys Res Commun. 2020. doi:10.1016/j.bbrc.2020.02.041 32087972

[92] Lee S‐H , So J , Shin D . Hepatocyte‐to‐cholangiocyte conversion occurs through transdifferentiation independently of proliferation in zebrafish. Hepatology. 2023;77:1198‐1210. doi:10.1097/hep.0000000000000016 36626626 PMC10023500

[93] Kitade M , Factor VM , Andersen JB , et al. Specific fate decisions in adult hepatic progenitor cells driven by MET and EGFR signaling. Genes Dev. 2013;27:1706‐1717. doi:10.1101/gad.214601.113 23913923 PMC3744728

[94] Pepe‐Mooney BJ , Dill MT , Alemany A , et al. Single‐cell analysis of the liver epithelium reveals dynamic heterogeneity and an essential role for YAP in homeostasis and regeneration. Cell Stem Cell. 2019;25:23‐38. doi:10.1016/j.stem.2019.04.004 e28 31080134 PMC6814390

[95] Anakk S , Bhosale M , Schmidt VA , Johnson RL , Finegold MJ , Moore DD . Bile acids activate YAP to promote liver carcinogenesis. Cell Rep. 2013;5:1060‐1069. doi:10.1016/j.celrep.2013.10.030 24268772 PMC3961013

[96] Zhao B , Wei X , Li W , et al. Inactivation of YAP oncoprotein by the Hippo pathway is involved in cell contact inhibition and tissue growth control. Genes Dev. 2007;21:2747‐2761. doi:10.1101/gad.1602907 17974916 PMC2045129

[97] Moya IM , Halder G . Hippo‐YAP/TAZ signalling in organ regeneration and regenerative medicine. Nat Rev Mol Cell Biol. 2019;20:211‐226. doi:10.1038/s41580-018-0086-y 30546055

[98] Cook D , Ogunnaike BA , Vadigepalli R . Systems analysis of non‐parenchymal cell modulation of liver repair across multiple regeneration modes. BMC Syst Biol. 2015;9:71. doi:10.1186/s12918-015-0220-9 26493454 PMC4618752

[99] Fujiyoshi M , Ozaki M . Molecular mechanisms of liver regeneration and protection for treatment of liver dysfunction and diseases. J Hepatobiliary Pancreat Sci. 2011;18:13‐22. doi:10.1007/s00534-010-0304-2 20607568

[100] Hammoutene A , Rautou P‐E . Role of liver sinusoidal endothelial cells in non‐alcoholic fatty liver disease. J Hepatol. 2019;70:1278‐1291. doi:10.1016/j.jhep.2019.02.012 30797053

[101] Hammoutene A , Biquard L , Lasselin J , et al. A defect in endothelial autophagy occurs in patients with non‐alcoholic steatohepatitis and promotes inflammation and fibrosis. J Hepatol. 2020;72:528‐538. doi:10.1016/j.jhep.2019.10.028 31726115

[102] Ito Y , Hosono K , Amano H . Responses of hepatic sinusoidal cells to liver ischemia‐reperfusion injury. Front Cell Dev Biol. 2023;11:1171317. doi:10.3389/fcell.2023.1171317 37082623 PMC10112669

[103] Schoen JM , Wang HH , Minuk GY , Lautt WW . Shear stress‐induced nitric oxide release triggers the liver regeneration cascade. Nitric Oxide. 2001;5:453‐464. doi:10.1006/niox.2001.0373 11587560

[104] Preziosi M , Okabe H , Poddar M , Singh S , Monga SP . Endothelial Wnts regulate β‐catenin signaling in murine liver zonation and regeneration: a sequel to the Wnt‐Wnt situation. Hepatol Commun. 2018;2:845‐860. doi:10.1002/hep4.1196 30027142 PMC6049069

[105] Duan J‐Li , Zhou Zi‐Yi , Ruan B , et al. Notch‐regulated c‐kit‐positive liver sinusoidal endothelial cells contribute to liver zonation and regeneration. Cell Mol Gastroenterol Hepatol. 2022;13:1741‐1756. doi:10.1016/j.jcmgh.2022.01.019 35114417 PMC9046233

[106] Saviano A , Henderson NC , Baumert TF . Single‐cell genomics and spatial transcriptomics: discovery of novel cell states and cellular interactions in liver physiology and disease biology. J Hepatol. 2020;73:1219‐1230. doi:10.1016/j.jhep.2020.06.004 32534107 PMC7116221

[107] Michalopoulos G . HGF and liver regeneration. Gastroenterol Jpn. 1993;28(4):36‐39. doi:10.1007/bf02782887. Suppl. discussion 53‐36. 7683618

[108] Yin C , Evason KJ , Asahina K , Stainier DYR . Hepatic stellate cells in liver development, regeneration, and cancer. J Clin Invest. 2013;123:1902‐1910. doi:10.1172/jci66369 23635788 PMC3635734

[109] Kitade M , Kaji K , Yoshiji H . Relationship between hepatic progenitor cell‐mediated liver regeneration and non‐parenchymal cells. Hepatol Res. 2016;46:1187‐1193. doi:10.1111/hepr.12682 26895456

[110] Houck KA , Cruise JL , Michalopoulos G . Norepinephrine modulates the growth‐inhibitory effect of transforming growth factor‐beta in primary rat hepatocyte cultures. J Cell Physiol. 1988;135:551‐555. doi:10.1002/jcp.1041350327 3165094

[111] Olsen PS , Poulsen SS , Kirkegaard P . Adrenergic effects on secretion of epidermal growth factor from Brunner's glands. Gut. 1985;26:920‐927. doi:10.1136/gut.26.9.920 2863199 PMC1432851

[112] Liu W‐H , Ren Li‐Na , Wang T , Navarro‐Alvarez N , Tang Li‐J . The involving roles of intrahepatic and extrahepatic stem/progenitor cells (SPCs) to liver regeneration. Int J Biol Sci. 2016;12:954‐963. doi:10.7150/ijbs.15715 27489499 PMC4971734

[113] Shang H , Wang Z , Song Y . Liver progenitor cells‐mediated liver regeneration in liver cirrhosis. Hepatol Int. 2016;10:440‐447. doi:10.1007/s12072-015-9693-2 26742763

[114] Lee UE , Friedman SL . Mechanisms of hepatic fibrogenesis. Best Pract Res Clin Gastroenterol. 2011;25:195‐206. doi:10.1016/j.bpg.2011.02.005 21497738 PMC3079877

[115] Dedhar S , Williams B , Hannigan G . Integrin‐linked kinase (ILK): a regulator of integrin and growth‐factor signalling. Trends Cell Biol. 1999;9:319‐323. doi:10.1016/s0962-8924(99)01612-8 10407411

[116] Martucci N , Michalopoulos GK , Mars WM . Integrin linked kinase (ILK) and its role in liver pathobiology. Gene Expr. 2021;20:201‐207. 33482930 10.3727/105221621X16113475275710 PMC8201652

[117] Hynes RO . Integrins: bidirectional, allosteric signaling machines. Cell. 2002;110:673‐687. doi:10.1016/s0092-8674(02)00971-6 12297042

[118] Wynn TA , Vannella KM . Macrophages in tissue repair, regeneration, and fibrosis. Immunity. 2016;44:450‐462. doi:10.1016/j.immuni.2016.02.015 26982353 PMC4794754

[119] Vannella KM , Wynn TA . Mechanisms of organ injury and repair by macrophages. Annu Rev Physiol. 2017;79:593‐617. doi:10.1146/annurev-physiol-022516-034356 27959618

[120] Sousa Da Silva RX , Weber A , Dutkowski P , Clavien P‐A . Machine perfusion in liver transplantation. Hepatology. 2022;76:1531‐1549. doi:10.1002/hep.32546 35488496

[121] Martins PN , Buchwald JE , Mergental H , Vargas L , Quintini C . The role of normothermic machine perfusion in liver transplantation. Int J Surg. 2020;82s:52‐60. doi:10.1016/j.ijsu.2020.05.026 32417462

[122] Lascaris B , De Meijer VE , Porte RJ . Normothermic liver machine perfusion as a dynamic platform for regenerative purposes: what does the future have in store for us? J Hepatol. 2022;77:825‐836. doi:10.1016/j.jhep.2022.04.033 35533801

[123] Hann A , Nutu A , Clarke G , et al. Normothermic machine perfusion‐improving the supply of transplantable livers for high‐risk recipients. Transpl Int. 2022;35:10460. doi:10.3389/ti.2022.10460 35711320 PMC9192954

[124] Li M , Fang F , Sun M , Zhang Y , Hu M , Zhang J . Extracellular vesicles as bioactive nanotherapeutics: an emerging paradigm for regenerative medicine. Theranostics. 2022;12:4879‐4903. doi:10.7150/thno.72812 35836815 PMC9274746

[125] Córdoba‐Jover B , Arce‐Cerezo A , Ribera J , et al. Cerium oxide nanoparticles improve liver regeneration after acetaminophen‐induced liver injury and partial hepatectomy in rats. J Nanobiotechnol. 2019;17:112. doi:10.1186/s12951-019-0544-5 PMC6822381 31672158

[126] Chen Y , Shi S , Li Bo , et al. Therapeutic effects of self‐assembled tetrahedral framework nucleic acids on liver regeneration in acute liver failure. ACS Appl Mater Interfaces. 2022;14:13136‐13146. doi:10.1021/acsami.2c02523 35285610

[127] Barreto Da Silva T , Dias EA , Cardoso LMDaF , Gama JFG , Alves LA , Henriques‐Pons A . Magnetic nanostructures and stem cells for regenerative medicine, application in liver diseases. Int J Mol Sci. 2023;24. doi:10.3390/ijms24119293 PMC10253131 37298243

[128] El Baz H , Demerdash Z , Kamel M , et al. Potentials of differentiated human cord blood‐derived unrestricted somatic stem cells in treatment of liver cirrhosis. Exp Clin Transplant. 2019;17:251‐258. doi:10.6002/ect.2017.0249 30346265

[129] Zhang J , Lu T , Xiao J , et al. MSC‐derived extracellular vesicles as nanotherapeutics for promoting aged liver regeneration. J Control Release. 2023;356:402‐415. doi:10.1016/j.jconrel.2023.02.032 36858264

[130] Zhang J , Gao J , Li X , et al. Bone marrow mesenchymal stem cell‐derived small extracellular vesicles promote liver regeneration via miR‐20a‐5p/PTEN. Front Pharmacol. 2023;14:1168545. doi:10.3389/fphar.2023.1168545 37305542 PMC10248071

[131] Zhu L , Wang Q , Guo M , et al. Mesenchymal stem cell‐derived exosomes in various chronic liver diseases: hype or hope? J Inflamm Res. 2024;17:171‐189. doi:10.2147/jir.S439974 38223423 PMC10788055

[132] Driscoll J , Wehrkamp C , Ota Yu , Thomas JN , Yan IK , Patel T . Biological nanotherapeutics for liver disease. Hepatology. 2021;74:2863‐2875. doi:10.1002/hep.31847 33825210

[133] Miyoshi T , Hiratsuka K , Garcia Saiz E , Morizane R . Kidney organoids in translational medicine: disease modeling and regenerative medicine. Dev Dyn. 2019;249(1):34‐45. 10.1002/dvdy.22 PMC6731163 30843293

[134] Kuse Y , Taniguchi H . Present and future perspectives of using human‐induced pluripotent stem cells and organoid against liver failure. Cell Transplant. 2019;28:160s‐165s. doi:10.1177/0963689719888459 31838891 PMC7016460

[135] Nie Y‐Z , Zheng Y‐W , Ogawa M , Miyagi E , Taniguchi H . Human liver organoids generated with single donor‐derived multiple cells rescue mice from acute liver failure. Stem Cell Res Ther. 2018;9:5. doi:10.1186/s13287-017-0749-1 29321049 PMC5763644

[136] Peng WC , Logan CY , Fish M , et al. Inflammatory cytokine TNFα promotes the long‐term expansion of primary hepatocytes in 3D culture. Cell. 2018;175:1607‐1619. doi:10.1016/j.cell.2018.11.012 e1615 30500539 PMC6497386

[137] Katsuda T , Kawamata M , Hagiwara K , et al. Conversion of terminally committed hepatocytes to culturable bipotent progenitor cells with regenerative capacity. Cell Stem Cell. 2017;20:41‐55. doi:10.1016/j.stem.2016.10.007 27840021

[138] He J , Cui H , Shi X , et al. Functional hepatobiliary organoids recapitulate liver development and reveal essential drivers of hepatobiliary cell fate determination. Life Medicine. 2022;1:345‐358. doi:10.1093/lifemedi/lnac055 PMC11749142 39872746

[139] Sun L , Hui L . Progress in human liver organoids. J Mol Cell Biol. 2020;12:607‐617. doi:10.1093/jmcb/mjaa013 32236564 PMC7683012

[140] Zhang K , Zhang L , Liu W , et al. In vitro expansion of primary human hepatocytes with efficient liver repopulation capacity. Cell Stem Cell. 2018;23:806‐819. doi:10.1016/j.stem.2018.10.018 e804 30416071

[141] Feng S , Wu J , Qiu W‐L , et al. Large‐scale generation of functional and transplantable hepatocytes and cholangiocytes from human endoderm stem cells. Cell Rep. 2020;33:108455. doi:10.1016/j.celrep.2020.108455 33296648

[142] Masuda Y , Yoshizawa K , Ohno Y , Mita A , Shimizu A , Soejima Y . Small‐for‐size syndrome in liver transplantation: definition, pathophysiology and management. Hepatobiliary Pancreat Dis Int. 2020;19:334‐341. doi:10.1016/j.hbpd.2020.06.015 32646775

[143] Søreide JA , Deshpande R . Post hepatectomy liver failure (PHLF)—Recent advances in prevention and clinical management. Eur J Surg Oncol. 2021;47:216‐224. doi:10.1016/j.ejso.2020.09.001 32943278

[144] Yuan X , Wu J , Sun Z , et al. Preclinical efficacy and safety of encapsulated proliferating human hepatocyte organoids in treating liver failure. Cell Stem Cell. 2024. doi:10.1016/j.stem.2024.02.005 38458193

[145] Sampaziotis F , Justin AW , Tysoe OC , et al. Reconstruction of the mouse extrahepatic biliary tree using primary human extrahepatic cholangiocyte organoids. Nat Med. 2017;23:954‐963. doi:10.1038/nm.4360 28671689

[146] Tysoe OC , Justin AW , Brevini T , et al. Isolation and propagation of primary human cholangiocyte organoids for the generation of bioengineered biliary tissue. Nat Protoc. 2019;14:1884‐1925. doi:10.1038/s41596-019-0168-0 31110298

[147] Sampaziotis F , Muraro D , Tysoe OC , et al. Cholangiocyte organoids can repair bile ducts after transplantation in the human liver. Science. 2021;371:839‐846. doi:10.1126/science.aaz6964 33602855 PMC7610478

[148] Tandon R , Froghi S . Artificial liver support systems. J Gastroenterol Hepatol. 2021;36:1164‐1179. doi:10.1111/jgh.15255 32918840

[149] Saliba F , Bañares R , Larsen FS , et al. Artificial liver support in patients with liver failure: a modified DELPHI consensus of international experts. Intensive Care Med. 2022;48:1352‐1367. doi:10.1007/s00134-022-06802-1 36066598

[150] Starokozhko V , Groothuis GMM . Challenges on the road to a multicellular bioartificial liver. J Tissue Eng Regen Med. 2018;12:e227‐e236. doi:10.1002/term.2385 27943623

[151] Larsen FS . Artificial liver support in acute and acute‐on‐chronic liver failure. Curr Opin Crit Care. 2019;25:187‐191. doi:10.1097/mcc.0000000000000584 30672818

[152] Feng L , Wang Yi , Fu Yu , Li T , He G . Stem cell‐based strategies: the future direction of bioartificial liver development. Stem Cell Rev Rep. 2024;20:601‐616. doi:10.1007/s12015-023-10672-5 38170319

[153] Shi X‐L , Gao Y , Yan Y , et al. Improved survival of porcine acute liver failure by a bioartificial liver device implanted with induced human functional hepatocytes. Cell Res. 2016;26:206‐216. doi:10.1038/cr.2016.6 26768767 PMC4746613

[154] Wang Y , Zheng Q , Sun Z , et al. Reversal of liver failure using a bioartificial liver device implanted with clinical‐grade human‐induced hepatocytes. Cell Stem Cell. 2023;30:617‐631. doi:10.1016/j.stem.2023.03.013 e618 37059100

